# Molecular mechanisms associated with microbial biostimulant-mediated growth enhancement, priming and drought stress tolerance in maize plants

**DOI:** 10.1038/s41598-022-14570-7

**Published:** 2022-06-21

**Authors:** Motseoa Lephatsi, Lerato Nephali, Vanessa Meyer, Lizelle A. Piater, Nombuso Buthelezi, Ian A. Dubery, Hugo Opperman, Margaretha Brand, Johan Huyser, Fidele Tugizimana

**Affiliations:** 1 Department of Biochemistry, University of Johannesburg, Auckland Park, Johannesburg, 2006 South Africa; 2 School of Molecular and Cell Biology, University of the Witwatersrand, WITS, Private Bag 3, Johannesburg, 2050 South Africa; 3 International Research and Development Division, Omnia Group, Ltd, Johannesburg, 2021 South Africa

**Keywords:** Plant sciences, Abiotic, Drought, Biochemistry, Metabolomics

## Abstract

Microbial-based biostimulants are emerging as effective strategies to improve agricultural productivity; however, the modes of action of such formulations are still largely unknown. Thus, herein we report elucidated metabolic reconfigurations in maize (*Zea mays*) leaves associated with growth promotion and drought stress tolerance induced by a microbial-based biostimulant, a *Bacillus* consortium. Morphophysiological measurements revealed that the biostimulant induced a significant increase in biomass and enzymatic regulators of oxidative stress. Furthermore, the targeted metabolomics approach revealed differential quantitative profiles in amino acid-, phytohormone-, flavonoid- and phenolic acid levels in plants treated with the biostimulant under well-watered, mild, and severe drought stress conditions. These metabolic alterations were complemented with gene expression and global DNA methylation profiles. Thus, the postulated framework, describing biostimulant-induced metabolic events in maize plants, provides actionable knowledge necessary for industries and farmers to confidently and innovatively explore, design and fully implement microbial-based formulations and strategies into agronomic practices for sustainable agriculture and food production.

## Introduction

Drought stress is increasingly diminishing yields of important cereals by over 10%^1^ and it is still the main limiting factor of food production in numerous countries^2^ affecting several crop plants such as maize (*Zea mays* L.)^3^. Water deficit negatively impacts plant growth and development by inducing an array of changes at molecular and cellular levels, translated into alterations in plant physiology and morphology^4^. Under drought stress, plants perceive the stress signals through receptors and sensors such as histidine kinases (HKs) and receptor-like kinases (RLKs)^5,6^. This triggers a generic signal transduction pathway that leads to the production of secondary messengers and the activation of a phosphorylation cascade that targets proteins involved in stress defence gene regulation^7^.

Early reaction signals have been identified, and these include increased cytosolic calcium (Ca^2+^), reactive oxygen species (ROS), and activation of the mitogen-activated protein kinase (MAPK) cascades which show cross-talks with other signalling molecule such as phytohormones (*e.g*. abscisic acid)^8^. Drought stress signals then induce the expression of downstream genes including late embryogenesis abundant (LEA) class genes (*RD29B, RAB18*) and functional gene products such as proline and glycine betaine^9^. These stress-regulated genes and their products play key roles in drought stress responses and tolerance by regulating cellular and physiological events such as osmolyte accumulation, membrane protection, ROS scavenging and stomatal closure through their translation into functional proteins. The natural drought stress responses mounted by plants are however not always sufficient to ensure plant survival under drought stress conditions. To overcome this, several approaches have been employed; and recently, an attention has been drawn to the use of biostimulants as a sustainable strategy.

The incorporation of biostimulant formulations and programs in the agriculture industry holds promise to sustainably improve crop productivity, considering that modern agriculture is facing a massive increase in demand due to twin pressures of an increasing population and environmental deterioration^10,11^. Additionally, climate change calamities have an impact on all facets of plant development, posing a significant challenge for developing sustainable agriculture. Plant growth-promoting rhizobacteria (PGPR)-based formulations form a category of microbial biostimulants that have captured the attention of the agricultural industry. PGPR are naturally predominant in the root systems of plants, and have co-evolved with the soil over millennia. These bacteria exhibit beneficial characteristics on plant yield and protection from adverse environmental conditions^12,13^.

Despite ongoing efforts made in studying and understanding the effects of biostimulants on plants, the underlying biostimulant-induced molecular and cellular events for plant growth promotion and stress resilience remain enigmatic. This knowledge gap in lack of fundamental understanding of modes of action of biostimulants hampers the novel formulation of biostimulants and the implementation of these products into agronomic practices^14^. In this work, we report an elucidation of metabolic alterations and molecular changes induced by a microbial biostimulant in maize plants. Interrogating maize metabolism, through the lenses of *omics* sciences, would enable the decoding of the language of cells at a molecular level. Such insights advance the understanding of regulatory network rules and mechanistic events at cellular and chemical space of maize responses to biostimulants, which, in turn, provides greater impetus for translation of fundamental knowledge to actionable programs in the field^7^.

In our previous untargeted metabolomics work, we reported the elucidation of a global metabolic landscape of maize leaves in response to a microbial biostimulant, under well-watered and drought conditions^15^. The study revealed alterations in a wide spectrum of both primary and secondary metabolites, including tricarboxylic acid (TCA) intermediates, amino acids, lipids and phenolics. Furthermore, this untargeted metabolomics work postulated that the application of microbial biostimulant conferred enhanced drought resilience to maize plants via altering key metabolic pathways involved in drought resistance mechanisms such as the redox homeostasis, osmoregulation, energy production and membrane remodelling^15^. Thus, as a follow-up and to further describe the postulated hypothetical framework on the global metabolic landscape of maize treated with a microbial biostimulant^15^, reported herein is a targeted metabolomics study, focusing on the key metabolic classes reported in the previous study *i.e.* amino acids, phytohormones and phenolics. The targeted analyses were complemented with gene expression and global DNA methylation analyses, to unravel metabolic events that fundamentally explain the effects of a consortium of *Bacillus* strains on maize plants under normal and drought conditions. The findings herein, therefore, contribute towards the generation of a fundamental knowledgebase describing the molecular mechanisms underlying the biostimulant effects on plants. Such insights are necessary for the advancement of the biostimulant industry and sustainable global food security.

## Results and discussion

The microbial biostimulant used in this study was a consortium of five *Bacillus* strains, referred hereafter to as simply PGPR. The study was designed as a targeted (quantitative) metabolomics approach, complemented with differential gene expression and global DNA methylation analysis. The study was designed to comprise six (6) treatment (T) groups as detailed in the experimental section. Briefly the six treatment groups include: (i) a control group of well-watered without PGPR (C), (ii) well-watered with PGPR (PGPR), (iii) mild drought with PGPR (MD-PGPR), (iv) severe drought with PGPR (SD-PGPR), (v) mild drought without PGPR (MD) and (vi) severe drought without PGPR (SD). The selected class of metabolites included amino acids, phytohormones, phenolic acids and flavonoids. Chemometric models, principal component analysis (PCA) and partial least squares–discriminant analysis (PLS-DA) revealed distinct drought- and biostimulant treatment-related sample groupings and allowed for the description of differential quantitative metabolic changes under the different treatments (Supplementary Figs. 1–10).

### Alterations in metabolic and gene expression profiles for growth promotion and defence priming

**Figure 1:**
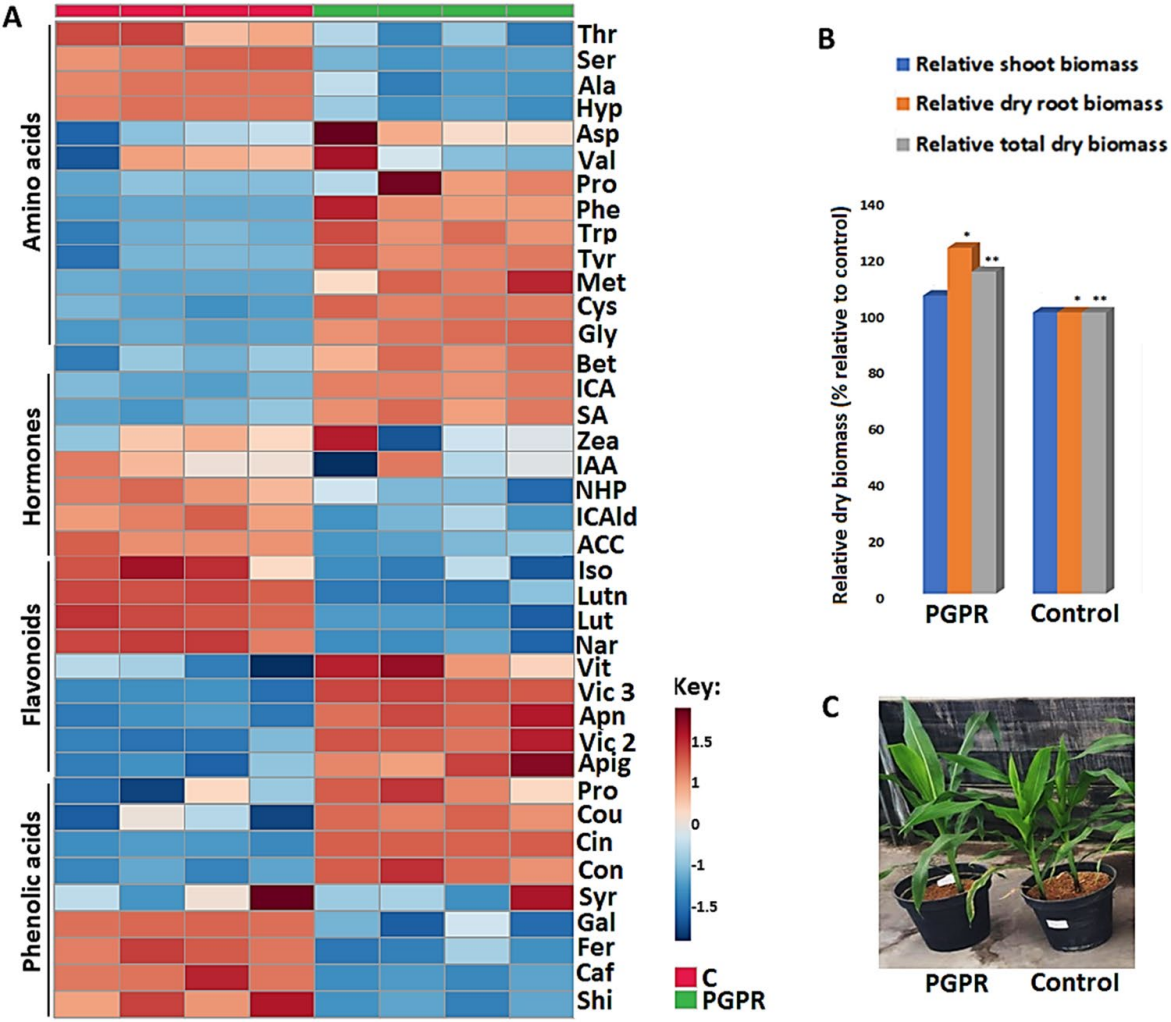
Quantitative and morphophysiological changes induced by PGPR under well-watered conditions. **(A**) Heatmap displays quantitative analysis of amino acids, phytohormones, flavonoids and phenolic acids, following hierarchal clustering of the samples. The heatmap was generated using the Pearson and Ward methods for distance measure and clustering measure respectively. The metabolite levels were clustered by compounds (rows) and biological replicates (columns) per treatment. Five plants were pooled into one biological replicate and four biological replicates were used. (**B**) Morphological changes: relative shoot, root and total dry biomass of the control and PGPR-treated plants with least significant difference (LSD) dry shoot biomass = 24.1, *LSD dry root biomass = 20.8, **LSD total dry biomass = 16.7. (**C**) Observable plant height and growth due to PGPR application at 4 weeks post emergence. (Metabolite abbreviations are defined in Supplementary Table 5).

Quantitative analyses of the selected amino acids revealed an increase in levels of glycine (Gly), cysteine (Cys), tyrosine (Tyr) (Fig. 1A) to be signatory markers of PGPR application in maize under normal (well-watered) conditions. These quantitative changes in amino acids (Fig. 1A) can be postulated as part of PGPR-induced remodelling of maize metabolism towards growth-promotion; phenotypically reflected in increased plant height and biomass (Fig. 1B,C). For example, Gly activates photosynthesis and enhances chlorophyll formation which in turn stimulate vegetative growth. Cysteine on the other hand occupies a central position in the plant metabolism where it plays an essential role of fixing inorganic sulfur in the photosynthetic primary sulfate assimilation^16^. Cysteine therefore acts as the only reduced sulfur donor molecule for the generation of numerous essential biomolecules such as methionine, glutathione, vitamin cofactors, iron-sulfur clusters and phytochelatins which are all essential for plant growth and development^17,18^. Accumulation of Tyr due to PGPR treatment under well-watered conditions was also observed in this study (Fig. 1A). Tyr is an aromatic amino acid (AAA) involved in the synthesis of proteins^19^, which can serve as a precursor for the biosynthesis of tocopherols such as vitamin E through its transamination into homogentisate from which vitamin E is synthesised^20,21^. Vitamin E can modulate ROS production, and therefore its accumulation may be beneficial to plant growth and survival by mitigating oxidative stress resulting from environmental cues.

Furthermore, amino acids generally play several roles in plants, from serving as basic building blocks of proteins to being crucial metabolites that interact with numerous branches of metabolism which stimulate plant growth^22^. Following degradation, amino acids’ carbon skeletons are generally converted into precursors or intermediates of the tricarboxylic acid (TCA) cycle—a central metabolic hub required for ATP production, contributing to mitochondrial metabolism and energy production in the form of ATP^23^. The energy produced from the TCA cycle can fuel a wide-range of energy-demanding biochemical processes that aid in plant growth and development such as gene expression, mobility and metabolism^24^. The proposed increase in ATP production induced by PGPR treatment through the amino acid accumulation can be postulated to be a form of plant growth promotion, phenotypically observed in Fig. 1B,C.

Quantitative measurement of selected phytohormones showed indole-3-carboxylic acid (ICA), salicylic acid (SA), indole-3-carboxaldehyde (ICAld), 1-aminocyclopropane-1-carboxylic acid (ACC), N-hydroxyphthalimide (NHP), indole-3-carboxylic acid (ICA) and zeatin (Zea) to be significantly altered by PGPR treatment in maize plants. PGPR led to increased levels of salicylic acid (SA) and indole-3-carboxylic acid (ICA) (Fig. 1A). These measured changes in phytohormone levels point to PGPR-induced reprogramming of hormonal networks towards growth promotion (Fig. 1B,C) and defence priming phenomenology. Phytohormones are signalling molecules produced in minuscule quantities that also regulate every aspect of plant growth and development, as well as adaptation under constantly changing environments^25,26^. SA enhances photosynthetic rate, resulting in increased energy production^27^, which is then utilised by biological processes governing plant growth and development. An increased accumulation of SA induced by PGPR treatment (Fig. 1A) can therefore be associated with enhanced plant growth due to SA’s ability to modulate specific plant physiological processes such as seed germination, vegetative growth and respiration. Additionally, SA enhances the photosynthetic rate, increasing plant energy production, which is utilised by biological processes governing plant growth and development^27,28^.

The observed PGPR-induced accumulation of ICA (Fig. 1A) could be linked to the catabolism of indole-3-acetic acid (IAA) into ICA, which has been recognised as a priming metabolite^29^. Auxins, primarily IAA, are endogenous plant hormones known for their regulatory role in plant growth and development such as root growth promotion. Previous studies have demonstrated how plants have evolved a complex system that regulates IAA levels, including its synthesis from the tryptophan-dependent pathway^30^. In plants, IAA can be catabolised via the non-decarboxylative pathways, with major degradation products being indole-3-carboxylic acid (ICA), indole-3-aldehyde (IAld), 2-oxindole-3-acetic acid (oxIAA), and indole-3-carbinol (I3C)^31^. Low concentrations of IAA generally stimulate root growth, and therefore the PGRP-induced catabolism of IAA into ICA (Fig. 1A) and other intermediate suggests a plant growth promotion mechanism employed by this PGPR-based biostimulant, via an increased root growth. This PGPR-induced root growth can also be postulated as part of a defence priming phenomenology resulting in the enhanced uptake of nutrients and water under limiting environmental stress conditions^32^. Furthermore, the accumulation of ICA could be correlated to the observed decreased levels of ICAld (Fig. 1A). Studies have reported that ICAld can be oxidised to ICA^33,34^.

The application of microbial biostimulant on maize plants lead also to a decrease in ACC levels under well-watered conditions (Fig. 1A). 1-aminocyclopropane-1-carboxylic acid (ACC), is an immediate precursor of ethylene, involved in the regulation of plant development^35^ and defence responses^36^. ACC is normally degraded by ACC deaminase into nitrogen and α-ketobutyrate. The latter can be converted to succinyl-CoA, a TCA cycle intermediate required for energy production. The measured decreased level of ACC in PGPR-treated maize plants (Fig. 1A) can be postulated to be linked to its degradation, providing a nitrogen source and energy for plant growth and development. Previous studies have reported the ability of certain PGPR to produce ACC deaminase enzyme which degrades ACC, resulting in shoot and root growth promotion, enhancing plant growth and development, with priming effects^37–40^.

Application of PGPR on maize plants under well-watered conditions also showed quantitative changes in secondary metabolites, particularly in selected flavonoids and phenolic acids including vicenin 2, apigetrin, apigenin, cinnamic acid, coniferyl alcohol, coumaric acid and caffeic acid (Fig. 1A) as signatory markers of PGPR treatment. The observed modifications in the secondary metabolism, induced by PGPR, suggest both plant growth promotion and priming phenomenology. Secondary (also termed specialized) metabolites are widely distributed in plants and are usually classified based on their biosynthetic pathways, and three major families are generally considered: alkaloids, terpenes/steroids and phenolics^41^. Flavonoids have been reported to have diverse functions that include control of respiration and photosynthesis^42^, antioxidant and chelating capacity^43^, and drivers of symbiosis between plants and rhizobacteria^44^. Thus, the observed increased levels of apigenin, apigetrin and vicenin in maize plants treated with PGPR (Fig. 1A) can be postulated to be underlying PGPR-induced metabolic reprogramming towards growth enhancement and defence priming.

Furthermore, a decrease in the level of a flavonoid naringenin was observed in PGPR-treated plants (Fig. 1A). Naringenin is a general precursor for the synthesis of isoflavones, flavones and flavonols through the action of flavone synthases^45^. The observed (PGPR-induced) decrease in naringenin levels is possibly due to its conversion into flavones apigenin and its glycosides (vicenin-2, vicenin-3, and apigetrin) (Fig. 1A). Pillai and Swarup^46^ reported the ability of *Pseudomonas putida* (PGPR) to induce a catabolism of naringenin and quercetin. The PGPR-induced reprogramming of flavonoid profiles has been previously reported, with a correlation to plant growth and defences^47–49^. Furthermore, PGPR treatment led to an accumulation of phenolic acids such as coumaric acid, cinnamic acid and coniferyl alcohol (Fig. 1A). Previous studies have shown an increase in the levels of phenolic acids induced by PGPR^50–53^. Phenolic acids mediate plant growth and reproduction by influencing physiological processes including cell division, seed germination and synthesis of photosynthetic pigments^54–57^.

We can thus postulate that the observed accumulation of the various phenolic acids induced by PGPR treatment (Fig. 1A) indicates a PGPR-induced enhancement of plant growth and development through various physiological events. This was translated into increased biomass and the plant phenotype (Fig. 1B,C). Flavonoids and phenolic acids, have an antioxidant capacity, and their accumulation can inhibit the generation of ROS, through ROS scavenging and hindering of the production of ROS producing enzymes. The accelerated accumulation of these secondary metabolites can inhibit ROS inside plant cells, thus maintaining a redox state inside plant cells. Studies have reported on the antioxidant activity (reducing agents, quenchers of singlet oxygen formation and free radical scavengers) of these compounds related to plant adaptation under abiotic stress^58,59^. PGPR-induced accumulation of these secondary metabolites serves as a priming mechanism to pre-condition the plant antioxidant system, resulting in a more robust defence system following stress cues. Priming or pre-conditioning (of plant defences and adaptive mechanisms) as stress memory is a state in which plants are rendered more resistant to subsequent stresses, displaying faster and more efficient defence and protective responses^60^. Previous studies have reported on the ability of PGPR to induce a reprogramming of secondary metabolites profiles^53,61^ and their ability to decrease oxidative stress in plant cells^62^, which resonate with our findings.

**Figure 2:**
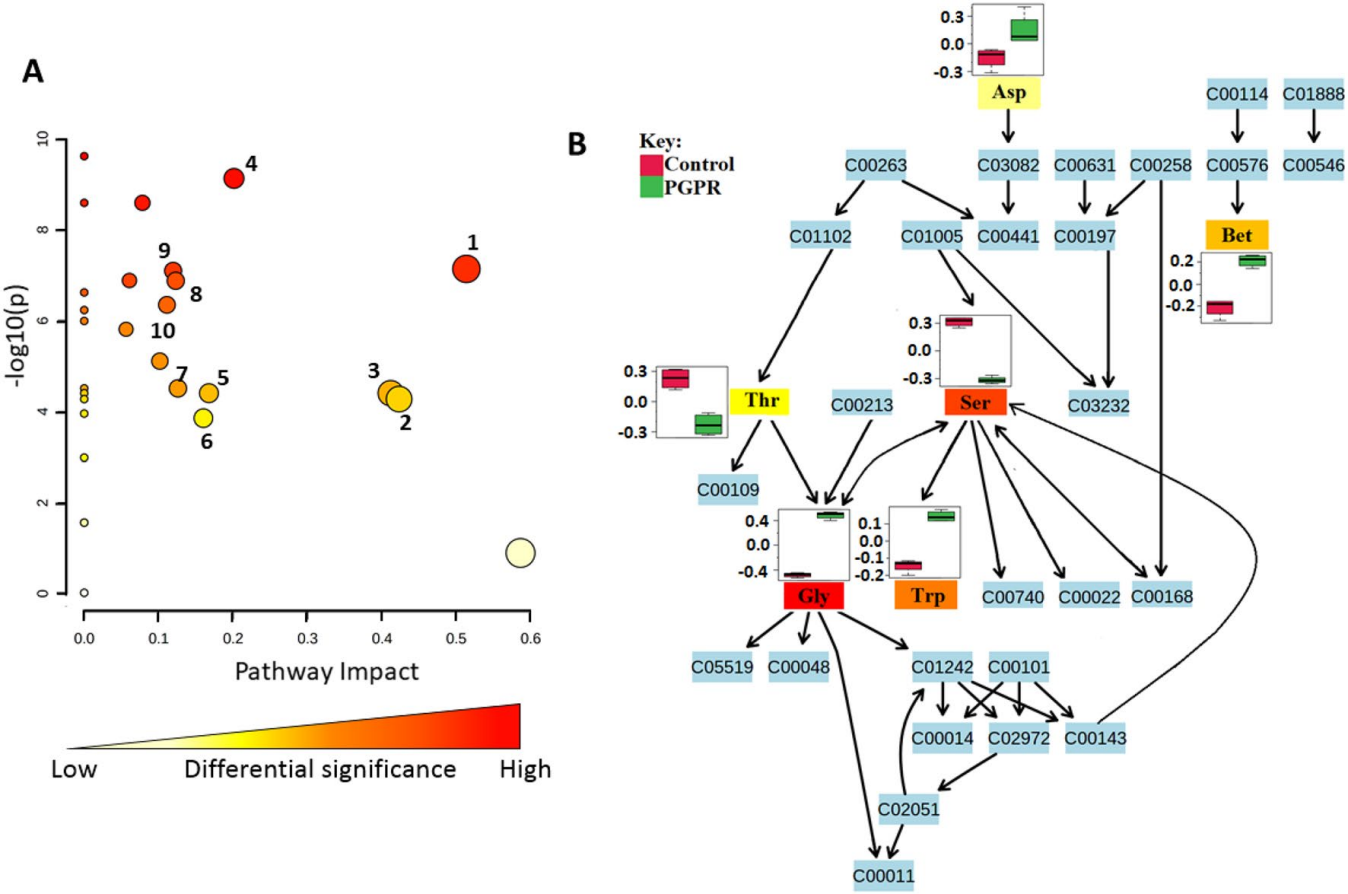
Metabolic pathways analysis revealing differentially altered pathways due to PGPR treatment. (**A**) A metabolome view indicating all the matched pathways arranged by *p*-values (pathway enrichment analysis) on the *y*-axis, and the pathway impact values (pathway topology analysis) on the *x*-axis, with the numbers corresponding to the mapped pathways listed in Supplementary Table S1. The node colour is based on the *p*-value and the node radius is defined by the pathway impact values. The latter is the cumulative percentage from the matched metabolite nodes. (**B**) Topology map of glycine, serine and threonine metabolism displaying altered amino acids in response to PGPR treatment. Abbreviations: Bet; Betaine, Asp; Aspartic acis, Thr; Threonine, Ser; Serine, Gly; Glycine, and Trp; Tryptophan. As mentioned in the experimental section, this infographics (with both A and B components) is an output from pathway analyses performed using the MetaboAnlyst bioinformatics suite^102^.

**Figure 3:**
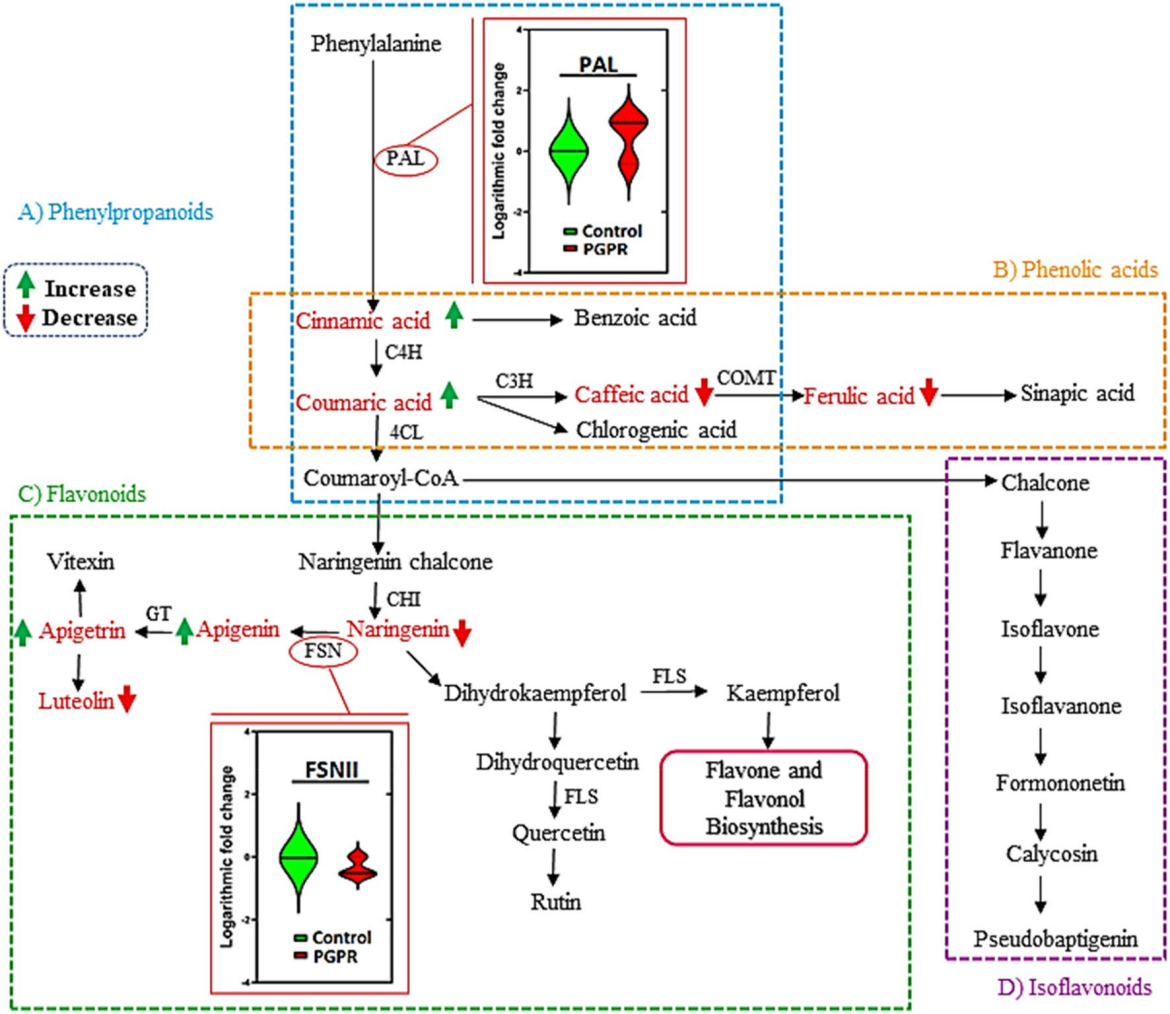
General phenylpropanoid pathway branching into phenolic acids, flavonoids and isoflavonoids biosynthesis. The pathways show the quantitative changes of phenolic acids and flavonoids together with differential gene expression of key genes (*PAL* and *FNS*) induced by PGPR treatment in well-watered plants and the inter-connection of the metabolites in different pathways.

Based on quantitative pathway analysis of the differentially abundant metabolites (in maize leaves), ten biological pathways were the most statistically altered by the application of the microbial biostimulant. These include biosynthesis pathways and several primary and secondary metabolism pathways such as Gly, Ser and Thr metabolism, flavonoid biosynthesis, and Phe, Tyr and Trp biosynthesis (Fig. 2A; Supplementary Table 1). The Gly, Ser and Thr pathway (Fig. 2B) plays a key role in the synthesis of additional amino acids such as lysine (Lys), Thr, Met, and isoleucine (Ile)^23^, and flavonoid biosynthesis. Phe, Tyr and Trp biosynthesis pathways, on the other hand, contribute to the synthesis of intermediate compounds that act as precursors for secondary metabolism (general phenylpropanoid pathway) which, in turn, plays a fundamental role in the plant-environmental interactions^63,64^. Additionally, the phenylpropanoid and flavonoid biosynthesis pathways (Fig. 3) play significant roles in the priming phenomenology. The measured alterations in secondary metabolite levels were further explained by PGPR-induced changes in gene expression profiles (Fig. 3). PGPR induced the upregulation of *phenylalanine ammonia lyase (PAL)* (6.3-fold) and a decrease in the expression level of *flavone synthase (FSNII)* (0.6-fold) (Fig. 3). Both *PAL* and *FSNII* genes are involved in the secondary metabolism. PAL is a key gateway enzyme that links the primary and secondary metabolism, mainly via the phenylpropanoid pathway, which branches into a network of other pathways. This enzyme catalyses the deamination of phenylalanine giving rise to cinnamic acid, which then serves as precursor for the biosynthesis of other phenylpropanoids^65^. FSNII, on the other hand, catalyses the direct conversion of flavanones (the precursors to all the major flavonoid classes) into flavones^66^.

**Figure 4:**
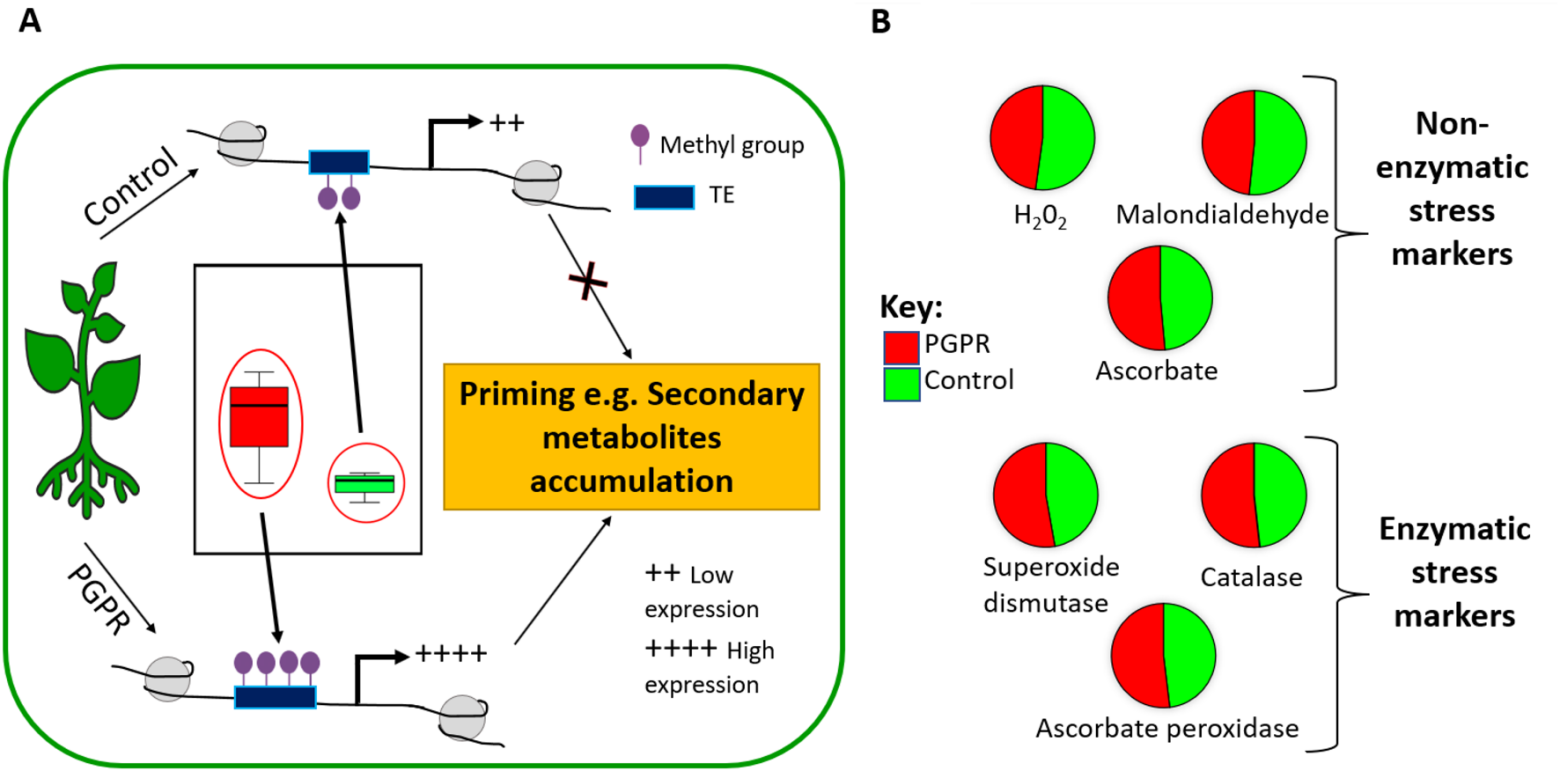
PGPR-induced differential changes under normal conditions, the pre-challenge phase. (**A**) Proposed model of PGPR-induced DNA methylation changes and inducing the biosynthesis of secondary metabolites. Genomic DNA from maize leaves in control and PGPR conditions was used to determine the relative global DNA methylation using ELISA. The Kruskal–Wallis test reported no statistical significance between the two groups; however, since methylation levels directly influence the genome, this change suggests that the reported difference may be biologically significant. (**B**) Quantitative changes (Supplementary Table S2) in antioxidant stress markers (non-enzymatic and enzymatic) of PGPR-treated and control plants. Abbreviations: C, control; PGPR, plant growth-promoting rhizobacteria.

The differential expression of *PAL* and *FSNII* indicates that PGPR activates the expression and translation of these key enzymes involved in the activation of the secondary metabolism pathways. This activation subsequently drives the biosynthesis of additional secondary metabolites, thus establishing a primed state of readiness in maize plants through an enhanced antioxidant capacity and other cellular and biochemical events. Furthermore, evaluating the DNA methylation changes via global quantification of 5-methylcytosine (5-mC), global DNA methylation levels were higher in PGPR-treated plants than in naïve plants. PGPR-treated plants showed an increase of 2.4-fold (33.3%) of global DNA methylation when compared to the naïve plants (Fig. 4A). This suggests that PGPR-induced rewiring of the maize metabolism is a multi-layered phenomenon: from gene regulation (at epigenetic level) to alterations in the metabolite levels. Remodelling of DNA methylation can also point to priming and cellular memory^67,68^.

Evidence of epigenetic regulation as one of the key mechanisms in the priming phenomenology has been reported in various studies^69^. A recent study by De Palma et al.^70^ described how *Trichoderma harzianum* T22 induced epigenomic modifications (hypermethylation) in tomato roots. Yang et al.^71^ reported that DNA methylation regulates the expression of key genes involved in the biosynthesis of phenolic acids in *Salvia miltiorrhiza.* The accumulation of these secondary metabolites can pre-condition the plant through various mechanisms such as the establishment of antioxidant machinery. Thus, with the background of this emerging literature on epigenetic regulation of the priming phenomenology, we can hypothetically postulate that the observed PGPR-induced changes in DNA methylation levels, affect the expression of various genes, and the latter could span genes involved in the biosynthesis of secondary metabolites, possibly such as *PAL* and *FSNII* (Fig. 3) and other genes involved in the priming events. The reported differential global DNA methylation (Fig. 4), as well as gene expression changes, may be retained and carried forward to subsequent generations as a priming memory^72^. This memory phenomenon is manifested under stress conditions (see the subsequent section).

These PGPR-induced molecular changes (at epigenetic, genetic and metabolic levels; Figs. 1, 2, 3 and4A) were translated into a functional alteration of maize physiology. Assessing enzymatic and non-enzymatic antioxidant markers revealed an enhanced production of antioxidants. PGPR treatment led to an accumulation of leaf ascorbate (AsA), superoxide dismutase (SOD) and ascorbate peroxidase (APX) (Fig. 4B). These physiological changes reflect the PGPR-induced primed state (*i.e*., a preconditioned antioxidant detoxification system) of maize plants. The increase of these antioxidant markers signifies enhanced cellular detoxification of ROS species, as evidently shown by a decrease in hydrogen peroxide (H_2_O_2_) levels in PGPR treated plants (Fig. 4B). Various studies including the studies by Khan et al.^73^ and Yang et al.^74^, have shown that PGPR have a positive effect on the antioxidant capacity of various plant species. Thus, a biochemical framework emerging from these results (Figs. 1, 2, 3 and 4) indicates that the PGPR-based biostimulant induces a multi-layered reprogramming of maize metabolism towards growth promotion and priming. This metabolic remodelling leads to morphophysiological events including enhanced (1) root, shoot and leaf growth, (2) nutrient uptake, (3) energy production, (4) protein synthesis and (5) antioxidant capacity.

### Metabolic changes associated with enhanced plant resilience against drought conditions

**Figure 5:**
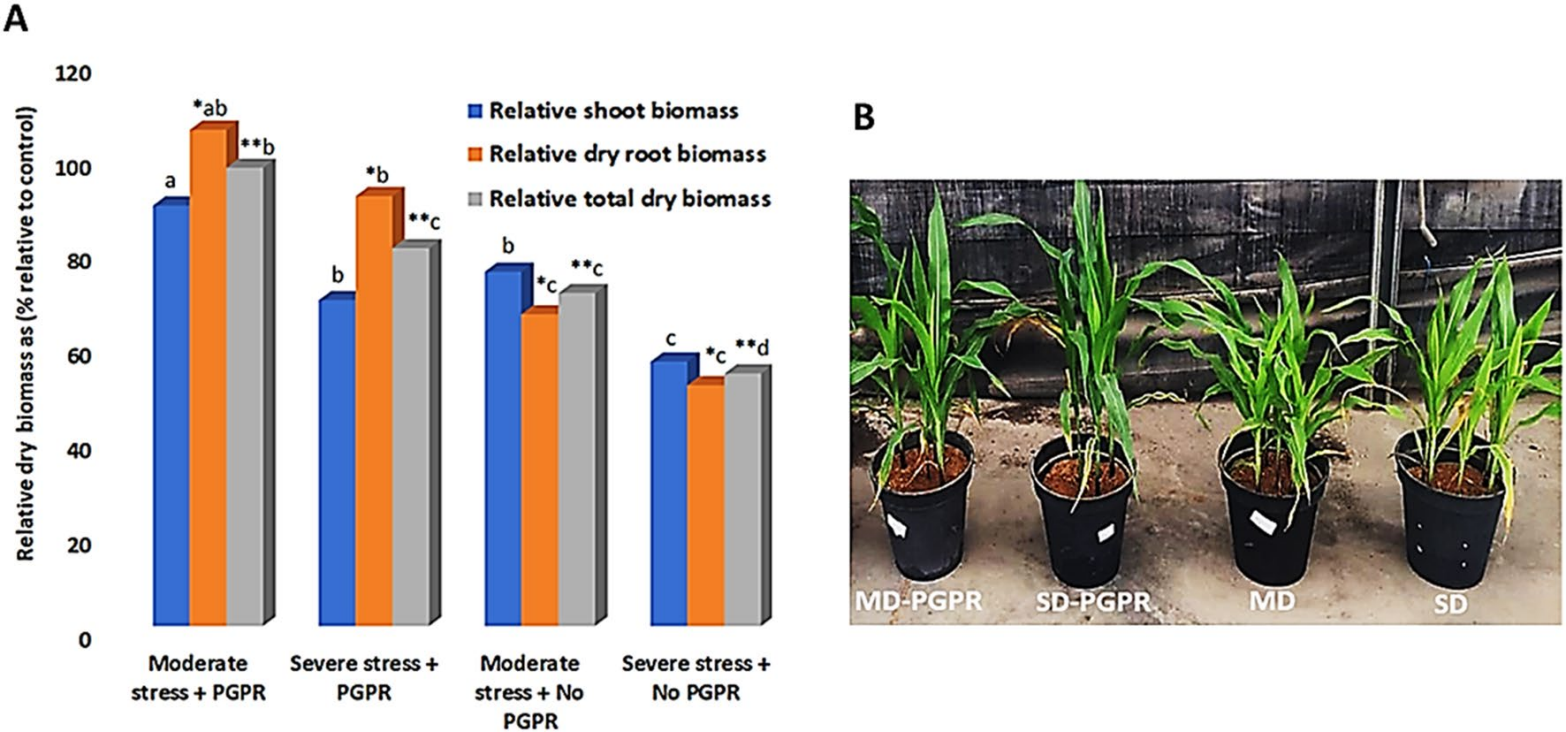
Morphophysiological changes in maize plants. Maize plants treated with PGPR and under drought (mild and severe) stress conditions. (**A**) Relative biomass changes and (**B**) phenotypic observations under the different treatments at 4 weeks post emergence. MD = mild (or moderate) stress; SD = severe stress conditions. Different letters indicate statistically significant differences (*p* ≤ 0.05); LSD dry shoot biomass = 24.1, *LSD dry root biomass = 20.8, **LSD total dry biomass = 16.7. Five plants were pooled into one biological replicate and four biological replicates were used.

Phenotypically, PGPR-treated and moderately stressed plants showed a 19%, 59% and 38% increase in the shoot, roots and total dry biomass, respectively, whereas the PGPR-treated and severely stressed plants showed a 23%, 78% and 49% increase in the shoot, roots and total dry biomass, respectively (Fig. 5A), when compared to the controls. This increase in biomass in PGPR-treated *vs*. naïve plants was also reflected in the differential plant height (Fig. 5B). These phenotypic observations suggest that PGPR induced better performance in maize plants under drought conditions, pointing to priming phenomenology, defined by the PGPR-induced metabolic and molecular remodelling.

**Figure 6:**
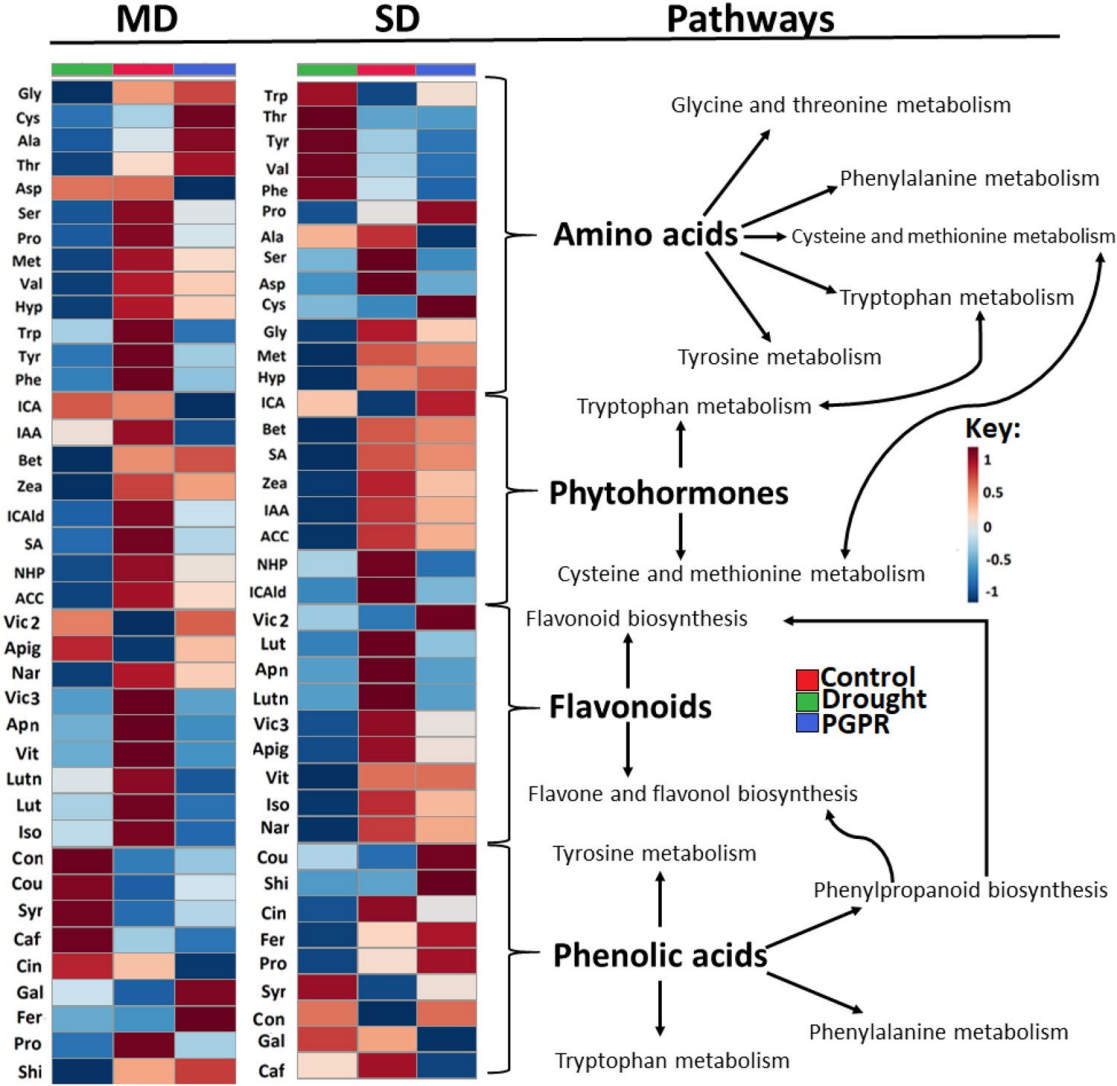
Quantitative analysis of amino acids, phytohormones flavonoids and phenolic acids. Heatmap hierarchical cluster analysis displaying metabolite abundances under control, mild /severe drought stress (MD,SD) and mild /severe drought stress conditions with PGRP treatment (MD-PGPR, SD-PGPR), together with differentially altered metabolic pathways**.** The heatmap was generated using the Pearson and Ward methods for distance measure and clustering measure respectively. The metabolite levels were clustered by compounds (rows) and averaged biological replicates (columns) per treatment. Five plants were pooled into one biological replicate and four biological replicates were used. Abbreviations: Mild drought, MD; and Severe drought, SD.

The morphophysiology of primed maize plants in the post-challenge phase (under drought conditions) is explained by the underlying PGPR-induced metabolic and molecular reprogramming (Figs. 1, 2, 3 and 4). Under both moderate and severe drought stress, Gly, Cys, and Bet levels were increased in PGPR-treated plants, whereas Tyr, Phe and Asp levels were decreased (Fig. 6).The elevated levels of amino acids such as Gly and Ser, could be linked to drought enhancing mechanisms such as stomatal regulation, osmotic adjustments and oxidative stress protection^15^. Differential metabolic changes were also observed in amino acids such as Thr and Trp in moderate and severe drought stress conditions. These differential metabolic changes show that the applied microbial biostimulant induces dynamic changes in the amino acid levels depending on the stress level.

**Figure 7:**
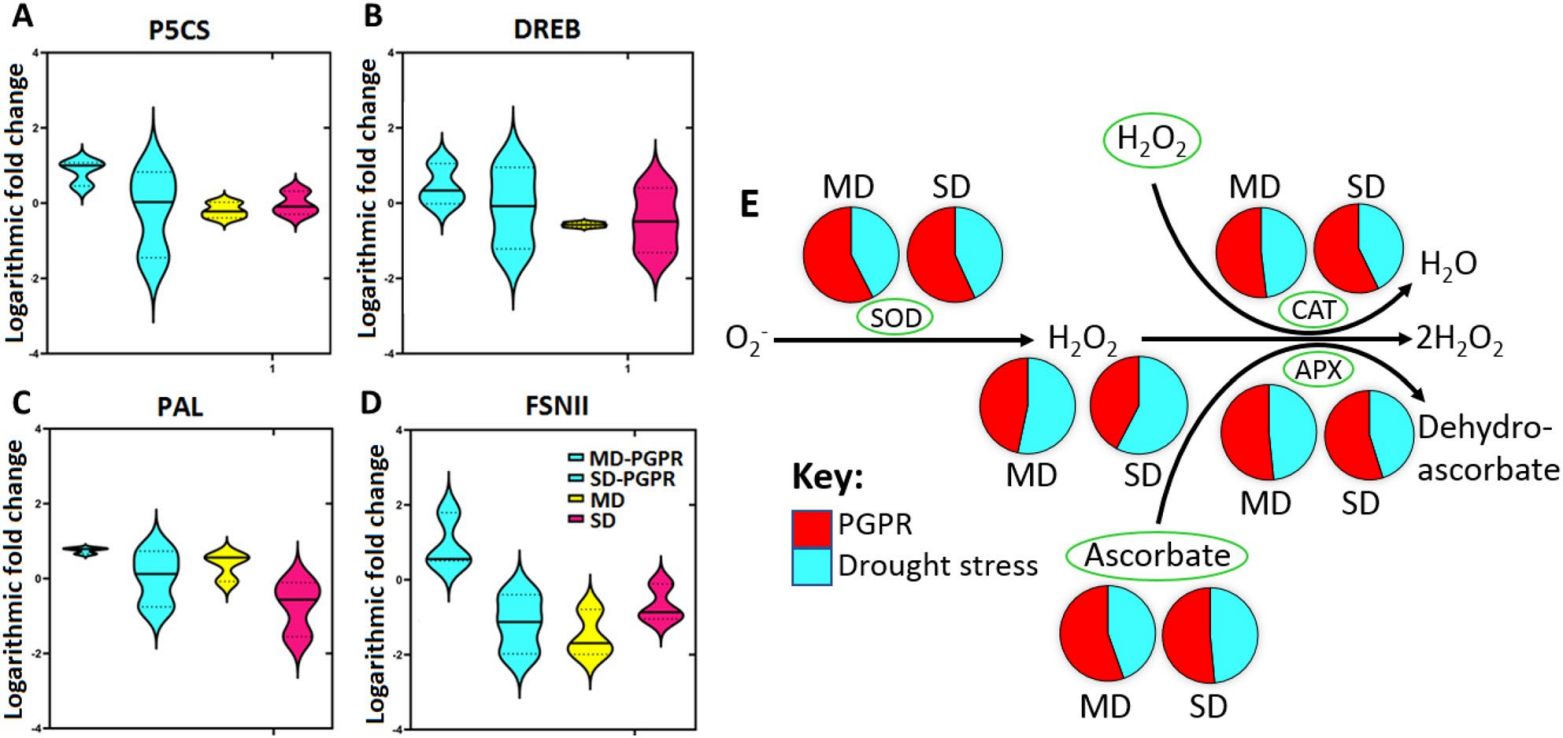
Gene expression profiles and changes in antioxidant stress markers in maize plants treated with PGPR, under stress conditions. Violin plots depict normalised gene expression expressed as logarithmic fold change of (**A**) *P5CS*, (**B**) *DREB*, (**C**) *PAL* and (**D**) *FSNII* under PGPR treatment, mild and severe drought stress (MD and SD respectively), and PGPR-primed drought stressed conditions. The relative quantification of each gene against reference genes (*EF1α* and *β-TUB*) was calculated, which was then used to calculate normalised gene expression. Violin plots show the distribution of data using density curves, which are overlaid by boxplots. The horizontal line within a violin plot represents the median. The lower and upper dotted lines show the 25th and 75th percentiles, respectively. (**E**) Antioxidant capacity pathway displaying different antioxidant markers circled in green (enzymatic and non-enzymatic) (Supplementary Table S2) under drought stress with and without PGPR treatment. C, control; PGPR, plant growth-promoting rhizobacteria; MD, mild drought and SD, severe drought.

Furthermore, the primed plants showed an increased level of the amino acid Pro under severe drought stress (Fig. 6). This was further evaluated and confirmed by the expression profile of *pyrroline-5-carboxyalate synthase (P5CS)*, a gene responsible for Pro biosynthesis^75,76^. The primed plants showed an upregulation of *P5CS* exhibiting 8.2- and 2.6-fold increase under MD and SD, respectively (Fig. 7A). Yoshiba et al.^77^ first reported an increase in proline content attributed to the upregulation of *P5CS* due to *Bacillus* inoculation under drought stress. Similarly, a recent study by Ghosh et al.^78^ reported on how *Pseudomonas putida* alleviates the effects of drought stress in *Arabidopsis thaliana* by drastically changing proline gene expression profiles. PGPR-induced accumulation of Pro is associated with improved drought tolerance via osmoprotection^79,80^. Additionally, Pro can act as a signalling molecule, free radical scavenger, cell redox balancer, source of carbon, nitrogen and energy, stabilizer for cellular structures and membranes, and an activator of detoxification pathways^81^.

Complementarily, to investigate the PGPR-induced osmoprotection, we also looked at *dehydration-responsive element-binding protein 2A (DREB2A)* gene expression in naïve and primed plants under drought conditions. PGPR-primed plants also exhibited an upregulated expression of *DREB2A* under both mild and severe drought stress conditions (8.4- and 5.7-fold, respectively) (Fig. 7B). *DREB* genes have been reported as the best studied group of transcription factors (TFs) involved in activating gene expression of many target genes responsible for controlling aspects such as osmoprotection under abiotic stress^82^. The modulation of the expression of drought responsive genes by *Bacillus* has been reported by Gagné-Bourque et al.^83^, in which plants inoculated with *Bacillus subtilis* displayed a higher accumulation of *DREB* and enhanced drought tolerance when compared to the non-inoculated plants. Thus, the up-regulation of *DREB2A* and *P5CS* observed in PGPR-primed stressed plants serves a confirmation that PGPR priming enhanced drought tolerance via osmoprotection avenues. Furthermore, the priming phenomenon was confirmed at the epigenetic level, by measuring the global DNA methylation. An increase in global DNA methylation levels was observed under both mild and severe drought stress conditions (3.7- and 6.4-fold; 9.2% and 21.5% respectively) in PGPR-primed plants (Supplementary Fig. 11). This increase in DNA methylation mimics control levels, suggesting a return to baseline expression and restored genomic integrity resulting in drought stress tolerance. Moreover, enhanced DNA methylation is considered as evidence of priming for enhanced defence response against abiotic stresses^84^*.*

Other metabolic changes observed under drought conditions include a decrease in the levels of aromatic amino acids, Tyr, Trp (mild drought only) and Phe in PGPR-treated plants (Fig. 6). The deamination of aromatic amino acids (Phe and Tyr) initiates the phenylpropanoid pathway catalysed by PAL enzyme (encoded by *PAL* gene) and tyrosine ammonia-lyase (TAL), key gateway enzymes that link primary and secondary metabolism. At the gene level, PGPR induced an upregulated expression of *PAL* and *FSNII* genes under mild drought stress conditions (4.6- and 12.7-fold, respectively) and a down-regulation of *FNSII* under severe drought stress conditions (0.6-fold) (Fig. 7C,D). The *FSNII* gene encodes the FSNII enzyme which is involved in the biosynthesis of flavones—a major class of flavonoids, stemming from the main phenylpropanoid pathway^65,85^. The differential expression of these genes can be correlated to metabolic reprogramming in PGPR-treated plants under mild and severe drought stress leading to differential changes in the levels of flavonoids and phenolic acids (Fig. 6). The primed plants showed an accumulation of naringenin and ferulic acid, and a decrease of isovitexin, luteolin, luteoside, coniferyl alcohol, cinnamic acid, caffeic acid and coumaric acid under mild drought stress (Fig. 6).

The accumulation of flavonoids and phenolic compounds has been linked to enhanced ROS scavenging through various mechanisms including inhibition of enzymes involved in ROS production and quenching^86,87^. Drought stress disturbs the balance between ROS generation and scavenging and thus accelerates ROS propagation which damages vital macromolecules (*e.g*. nucleic acids and proteins) and photosynthetic complexes, ultimately leading to cell death^88^. Thus, the increased levels of phenolic acid and flavonoids in PGPR primed plants can therefore contribute to the mitigation of oxidative stress induced by drought stress via non-enzymatic machinery. In addition to the non-enzymatic machinery, PGPR application was also shown to increase the activity of CAT, SOD and APX (enzymatic antioxidant machinery markers) under moderate and severe drought stress conditions (Fig. 7E). Therefore, these results evidently demonstrate that PGPR priming confers drought tolerance by enhancing the antioxidant machineries and the latter is illustrated by the differential profiles of flavonoids and phenolic acid (at the metabolic level), differential gene expression (*PAL* and *FSNII*—gene level) and increased enzymatic activity of antioxidant enzymes (at the physiological level).

Furthermore, apart from serving as precursors for phenolic compounds and their proteogenic function, aromatic amino acids also play critical roles in plant metabolism by serving as precursors for a variety of plant hormones. For example, Trp is involved in the synthesis of auxin-related hormones such as IAA, ICAld and ICA. IAA and ICAld were found in high content in PGPR-primed plants under mild drought and in low content in PGPR-primed plants under severe drought compared to naïve plants (Fig. 6). ICA, on the other hand, was decreased in PGPR-primed plants under mild drought stress (Fig. 6). Increased IAA levels have been positively associated with improved drought stress tolerance and delayed leaf senescence^89^, which can aid in the maintenance of the remobilization of stored nutrients ultimately resulting in improved crop yield and biomass^90^. Coincidingly, as phenotypically observed in this study, the biomass (root and shoot) of PGPR-primed plants was increased compared to naïve plants under drought stress (Fig. 5A,B).

Hormonal changes induced by PGPR also involved an increase in the levels of Zea, SA and ACC in PGPR-primed plants under mild drought stress conditions and a decrease in PGPR-primed plants under severe drought stress conditions compared to naïve plants (Fig. 6). Zeatin, ACC and SA are involved in growth and development processes such as cell division, root hair proliferation, stomatal conductance and regulation of water balance under drought stress^36^. The increased accumulation of cytokinins and SA by different PGPR strains under drought stress has been reported in previous studies^91,92^. Overall, the increase and decrease of the phytohormones observed in PGPR-primed plants under mild stress and severe stress, respectively, suggest that the effectiveness of PGPR priming in enhancing drought resistance vary depending on the intensity of the drought stress.

Under drought conditions, the significantly impacted pathways included Gly, Ser and Thr metabolism, Phe, Tyr and Trp biosynthesis, phenylpropanoid biosynthesis, flavone and flavonol biosynthesis amongst others (Fig. 6; Supplementary Table 3). Gly, Ser and Thr metabolism is involved in photorespiration, a process that plays a role in the regulation of growth-related types of machinery such as photosynthesis and osmoprotection^23^. Generally, amino acid metabolism is closely associated with carbon–nitrogen ratios, energy and sugar metabolism and secondary metabolism (*e.g*. Phe, Tyr and Trp biosynthesis)^22,93^. The phenylpropanoid biosynthesis, flavone and flavonol biosynthesis are mainly involved in the biosynthesis of secondary metabolites which play key roles in plant defence against multifarious environmental stresses^94^. As highlighted above, *PAL* and *FSNII* genes were upregulated in PGPR-treated plants under mild drought stress conditions (Fig. 7C,D), this confirms that indeed phenylpropanoid biosynthesis and flavone and flavonol biosynthesis were impacted by PGPR priming.

**Figure 8:**
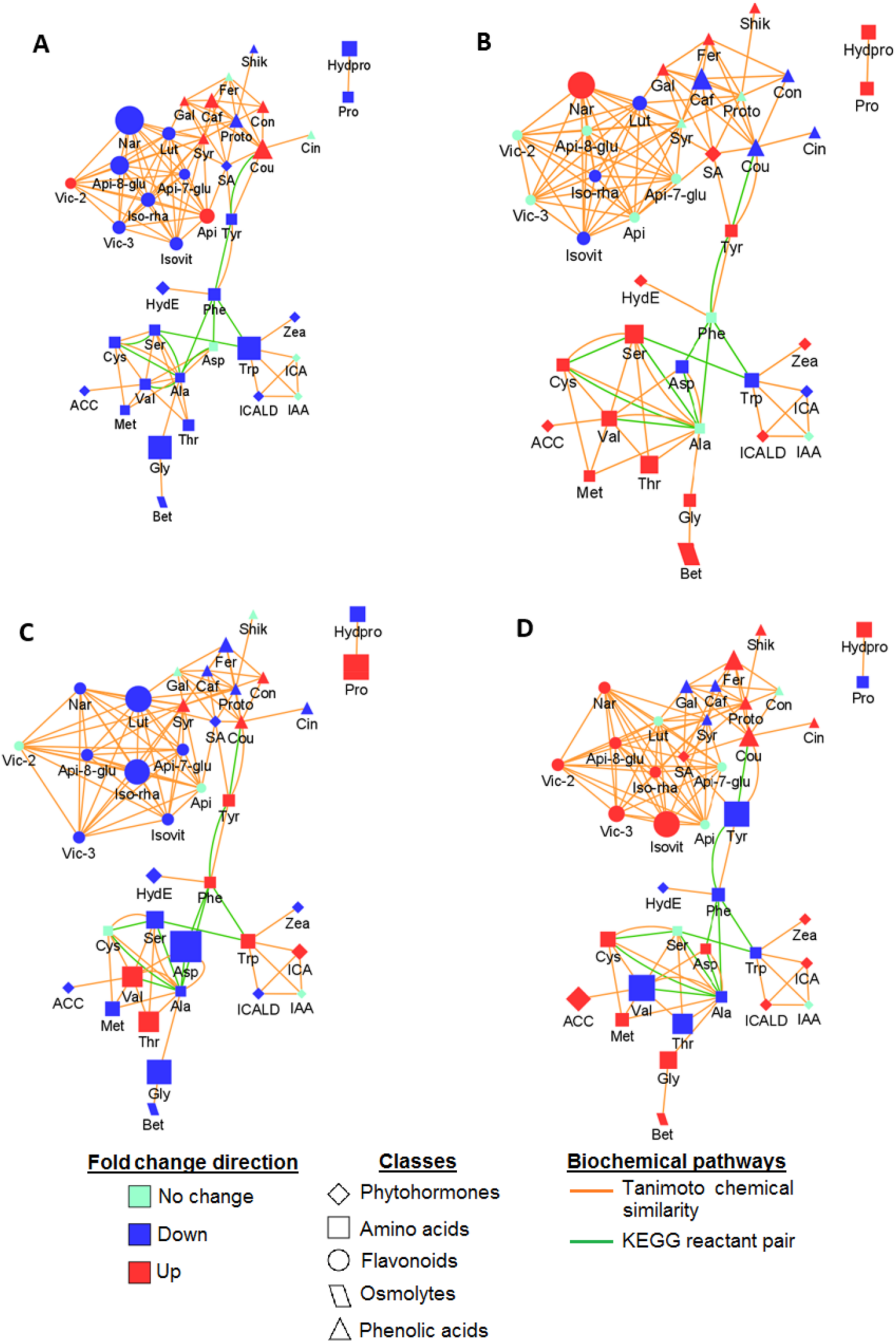
Metabolic network analysis highlighting differential metabolic interconnections. Different experimental conditions: (**A**) mild drought (**B**) mild drought—PGPR (**C**) severe drought (**D**) and severe drought—PGPR treatment. Green edges denote KEGG reactant pair links and orange edges symbolize chemical similarity. Metabolites found significantly up-regulated (*p* < 0.05) are given as red nodes and blue nodes give down-regulated metabolites. Node sizes reflect the magnitude of fold change.

Furthermore, correlation (metabolic) network analysis allowed the characterisation of the complex relationship in measured metabolites. The constructed metabolic network graphs (Fig. 8) depict relational patterns in the experimental data and identify altered graph neighbourhoods. These relationships do not depend on any predefined biochemical pathways and therefore allow for the characterisation of the molecular and cellular states induced by pathway interconnections under the stated experimental conditions^95^. This graphical representation depicts two major network clusters, phenylpropanoid-related metabolites (circles and triangles), and amino acids (squares), which are connected via Tanimoto chemical similarity and keg reactant pair (krp) interaction, indicating structural relatedness and biochemical relationship (Fig. 8). For instance, the krp interaction between Tyr and *p*-coumaric acid (Fig. 8) suggests an enzymatic conversion between the two metabolites^96^. This enzymatic conversion is catalysed by tyrosine ammonia lyase (TAL)—a shortcut pathway driving the phenylpropanoid biosynthesis^97^.

Thus, the generated network graphs showed a general decrease in the targeted amino acids in naïve plants (Fig. 8A and C), whereas a general increase was observed in the PGPR-primed plants (Fig. 8B and D). A previous study has shown that a decreased content of total amino acids in barley leaves under drought was associated with decreased absorption of nitrate by the roots, reduced translocation to the leaves and higher rates of photorespiration^98^. Moreover, it is expected that higher photorespiration will results in insufficient carbon available for the biosynthesis of amino acid thus resulting in decreased amino acid pool sizes^99^. In this logic, the findings of our study—the observed increase in amino acid content in primed plants—suggest normal photorespiration rate and increased nitrate absorption and translocation, one of the PGPR-mediated growth-promoting mechanisms, as reported also in previous studies^100^.

The metabolic network also showed multiple krp interactions between Ala and other amino acids such as Phe, Val, Cys, Ser and Asp (Fig. 8). This correlation highlights Ala as a central node, suggesting its potential involvement in the regulation of amino acid-related pathways^101^. For example, the biochemical relationship between Ala-Ser and Ala-Asp, suggest the regulatory role of Ala in Gly, Ser and Thr metabolism (Fig. 2 and Supplementary Tables 1 and 3)—a pathway involved in photorespiration. Ala is a known major amino donor in photorespiratory metabolism^98^ and its reduction was identified as an important marker for low CO_2_/high photorespiration. Based on the observation that Ala was decreased in the naïve plants whereas no change was observed in the levels of Ala in the PGPR-treated plants (Fig. 8), we can postulate that the PGPR priming prevents increased demands for the re-assimilation of photorespiratory-released NH_3_ and CO_2_, which is required under drought stress. Therefore, this illustrate that the microbial biostimulant induced a reconfiguration of maize metabolism via differential regulation of Gly, Ser and Thr metabolism to prevent high photorespiration, thus minimizing the metabolic costs.

**Figure 9:**
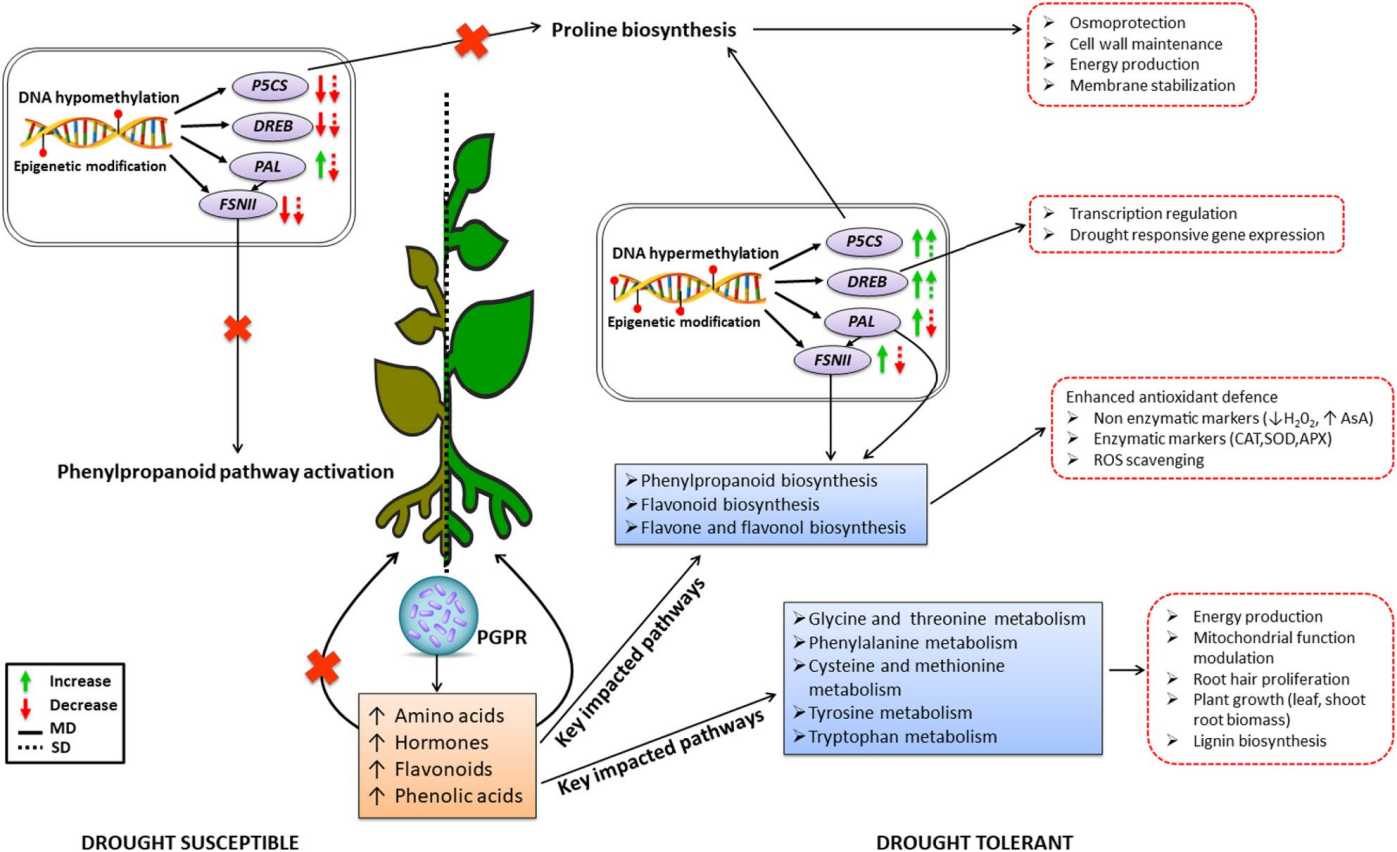
An elucidated model of biostimulant effects on maize plants. This summary diagram infographically depicts elucidated molecular mechanisms induced by PGPR in maize plants. PGPR-primed plants exhibit enhanced induction of drought stress responsive mechanisms such as increased pool of amino acids, hormones, flavonoids, phenolic acids, DNA hypermethylation and expression of key stress-responsive genes, resulting in drought stress tolerance.

In summary, this study provides an in-depth molecular understating of PGRP-induced differential morphophysiological, epigenetic, genetic and metabolic changes gravitating towards enhanced physiological events that govern growth promotion and drought stress tolerance (Fig. 9). In the pre-challenge phase, the key mechanisms include increased levels of amino acids, flavonoids, hormones, phenolic acids, antioxidant markers, DNA methylation, driving the expression of key genes which regulate priming and increased plant biomass. The observed changes when situated in the maize metabolome spanned key impacted pathways including Phe metabolism, Gly metabolism, and flavonoid biosynthesis associated with enhanced drought stress tolerance. PGPR-mediated drought stress tolerance mechanisms elucidated herein include enhanced (i) energy production facilitated by amino acid degradation into TCA intermediates, (ii) osmoregulation, (iii) cellular and membrane stabilisation, (iv) transcription regulation (enhanced expression of drought stress responsive defense genes), (v) antioxidant machinery, (vi) root hair proliferation and (vi) lignin biosynthesis. The model presented herein, through quantitative metabolite changes complemented with gene expression and global DNA methylation analyses, confirmatory complements the hypothetical framework reported by Nephali et al.^15^. A *Bacillus*-based biostimulant enhances growth and primes maize plants against abiotic factors by modulating metabolic pathways and gene regulation events. This mechanistic framework, explaining the modes of action of the microbial biostimulant, is a necessary and important step for the biostimulant industry, for devising a roadmap of formulations and biostimulant-based agricultural strategies for sustainable food production.

## Methods

### Chemicals

All the chemicals utilised for sample analyses were of analytical grade, highest purity and were obtained from different international providers. Methanol and acetonitrile were LC–MS grade from Romil (SPS, Cambridge, UK). Leucine enkephalin and formic acid from Sigma Aldrich (Munich, Germany). Water was purified using a Milli-Q Gradient A10 system (Siemens, Fahrenburg, Germany).

### Plant material, growth conditions and treatments

All the plant experiments were in compliance with relevant institutional, national, and international guidelines and legislation. Commercially available maize seeds (*Zea mays* L., Hybrid PAN 3Q-240, Pannar Seed, Greytown, KwaZulu-Natal, South Africa) was germinated and cultivated in a dedicated experimental greenhouse facility of the Omnia Nutriology division of the Omnia group (https://www.omnia.co.za). The plants were cultivated specifically for research purposes and appropriate permissions for experimental treatment, harvesting and metabolomic analyses were in place (Omnia Nutriology, South Africa). Maize plants were cultivated in 15 L pots (8 seeds per pot), each filled with slightly acidic (pH 5.2) sandy soil (17 kg). The pots were placed in a completely randomised design (CRD) order in a greenhouse at Omnia facilities in Sasolburg, Free-State province, South Africa. An experimental study design was developed in which all different conditions (control and treated), were described as treatment (T) (Supplementary Table 4). The control and treated groups consisted of control (C), mild drought without PGPR (MD), severe drought without PGPR (SD), well-watered with PGPR (PGPR), mild drought with PGPR (MD-PGPR) and severe drought with PGPR (SD-PGPR) respectively (Supplementary Table 4). A PGPR-based biostimulant formulation (Omnia Group Ltd, Bryanston, South Africa) containing five *Bacillus* strains was used in this study. The microbial formulation used in this study is BACSTIM100 (Omnia Group Ltd., Bryanston, South Africa), a consortium of five *Bacilli* strains (viable PGPR): two strains of *Bacillus licheniformis*, two stains of *Brevibacillus laterosporus* and one strain of *Bacillus amyloliquefaciens*. This microbial formulation is a spore forming product, commercially tested for stability (Omnia Group Ltd., Bryanston, South Africa). The PGPR-based biostimulant treatment diluted 100 times to 8 mL per pot was evenly applied at a rate of 2 L per hectare at planting stage using a micropipette in the furrow with the seed. Following the emergence, the 8 seedlings cultivated per pot were thinned to five healthy and uniform plants per pot. There were 4 pots per treatment and each pot was considered as a biological replicate. All pots were irrigated to 90% plant available water (PAW) to allow for good germination. Drought stress was imposed at the 2-leaf stage (2 weeks after emergence, WAE) by a withholding water method where the water level was allowed to drop to 50% PAW then maintained at that level for the mild drought stress group and dropped to 20% PAW for the severe drought conditions. The well-watered plants were maintained at the 90% PAW throughout the study. Greenhouse conditions that were measured daily included temperature (midday, 28 ± 3 °C and night 12 ± 2 °C), relative humidity (45 ± 8%) and (midday) light intensity (738 ± 41 µmole m^−2^ s^−1^).

### Plant material harvesting and metabolite extraction

Leaf tissue harvesting for all treatments and biological replicates was performed at two different time points; four and six weeks after emergence (WAE) and following mild and severe drought application referred to as (4 WAE and 6 WAE, respectively). Considering that leaves 1 and 2 were developed under well-watered conditions, only the plant leaves developed after drought stress application, *i.e*., leaves 3, 4, 5 were used in this study. As abovementioned, five plants were pooled into one biological replicate and four biological replicates were used per treatment. Thus, the plant leaves were cut off and rapidly immersed in liquid nitrogen to quench any possible enzymatic reactions and the leaf materials were then stored at − 80 °C. Extraction of metabolites was initiated by adding liquid nitrogen to the frozen leaf tissues and grinding them into a fine powder using a pestle and mortar. To avoid any chance of sample crossover, the pestle and mortar were cleaned (washed using dH_2_O and rinsed with 80% aqueous methanol) between samples. Following this, two grams (2 g) of the powder was weighed in a sterile Falcon tube and 20 mL of 80% cold methanol was added in a 1:10 m/v ratio. The mixture was then homogenised for 2 min using an Ultra-Turrax homogenizer and sonicated for 30 s using a probe sonicator (Bandelin Sonopuls, Germany) set at 55% power. The homogenizer and the probe were cleaned with 80% aqueous methanol between samples to avoid sample crossover. The resulting homogenates were centrifuged at 5100 rpm for 20 min at 4 °C. The supernatants were placed in 50 mL round-bottom flasks, evaporated to 1 mL at 55 °C using a Büchi Rotavapor R-200, and dried to completeness with a speed vacuum concentrator (Eppendorf, Merck, South Africa) set at 45 °C. Resuspension of the extracts was done using 500 µL LC–MS grade methanol : Milli-Q water (1:1, v/v), followed by filtration through 0.22 μm nylon filter into pre-labelled HPLC glass vials fitted with 500 µL inserts (Shimadzu, Johannesburg, South Africa). The filtered samples were then stored at 4 °C pending LC-ESI-QqQ-MS analysis.

### Preparation of standards and multiple reaction monitoring (MRM) method development

Amino acid -, phytohormone -, flavonoid—and phenolic acid standards used in this study were of ≥ 98% purity, obtained from Merck (Germany), Sigma (United States of America) and BDH (England) manufacturers. Thirty-eight metabolites including an internal standard (D-fluorophenylalanine) were selected and quantified, and these include amino acids, osmolytes, phytohormones, flavonoids and phenolics. The amino acids, phytohormones and osmolytes, flavonoids and phenolics working solutions were over the concentration ranges of 25–1000 µg/L, 8.7 × 10^−5^–43.7 nM, 10–1000 µg/L and 7.78–250 nM respectively. The working solutions were all prepared in 50% aqueous methanol (Romil, Cambridge, UK) and stored at 4 °C. The analysis was performed using a triple quadrupole mass spectrometry (LCMS-8050 (Shimadzu, Kyoto, Japan)), equipped with an electrospray ionization (ESI) source and ultra-high performance liquid chromatography (UHPLC) as a front-end. The MRM-MS method was used for absolute quantification of the targeted metabolite classes. MRM-MS conditions (Supplementary Table 5) were developed and optimisation was done by direct infusion into the ionization source (ESI); and the MRM optimization method tool (an integral component of LabSolutions LCMS software, Shimadzu Corporation) was used for collision energy (CE) optimisation for all the transitions, by collecting product ion scan data and finding the optimum CE for each transition.

### LC-ESI-QqQ-MS metabolite profiling by ultra-fast liquid chromatography

The prepared samples and standards were analysed on an UFLC system, equipped with a Shim-pack GIST C18 column (2 μm; 100 × 2.1 mm l.D) (Shimadzu, Kyoto, Japan), thermostatted at 40 °C. Chromatographic separation was achieved using a gradient elution system consisting of eluent A (MilliQ water with 0.1% formic acid) and eluent B (methanol with 0.1% formic acid) (Romil Chemistry, UK) at a constant flow rate of 0.2 mL min^−1^. Each metabolite class (amino acids, phytohormones, flavonoids and phenolics) had a specific elution gradient (Supplementary Table 6). The total chromatographic run time was 10, 40, 31 and 30 min; and injection volume 3, 1, 2, and 3 µL for amino acids, phytohormones, flavonoids and phenolic acids, respectively. The MRM-MS detection parameters developed and optimised (Supplementary Table 5) were then applied, and the MS conditions were as follows: nitrogen gas was used as a drying gas and a nebulising gas at flow rates of 10 L min^−1^ and 3 L min^−1^ respectively. The heating gas flow was set at 10 L min^−1^, interface temperature at 300 °C, interface voltage at 4 kV, DL temperature at 250 °C, and heat block temperature at 400 °C.

### Data analysis: processing, pre-treatment and chemometric analysis

LabSolution Quant Browser (Shimadzu, Kyoto, Japan) was used to process the LC-MRM-MS data acquired, from which the calibration curves were constructed to obtain the concentrations of the unknown samples expressed in ppb (for amino acids and phenolics) and nM (for hormones and flavonoids), which were then converted to ng/g to create a concentration data matrix. MetaboAnalyst 4.0^102^, a comprehensive web-based tool, was used for processing, analysing, visualising and interpreting the data. Prior to data analysis, MetaboAnalyst performs a data integrity check by assessing the data labels (class and concentration values), pair specifications, and detecting the presence of missing values or features using its integral algorithms. The tool has a default method of replacing missing values using small numbers (one-fifth of the minimum positive values of their corresponding variables in the data) which assumes that the missing values are a result of low signal intensity metabolites that are below the detection limit; however, no values were replaced in this study. Following missing values replacement is the data filtering option which aims to identify and remove low-quality data points that have an improbable contribution to the modelling of the data, thus improving performance and reducing the false discovery rate (FDR) for downstream statistical analysis^103,104^. Subsequent to the data integrity check, data normalisation, a data pre-treatment method was applied. The selected pre-treatment methods which were deemed appropriate for metabolite concentration adjustment in this study were transformation and *Pareto* scaling with no row-wise normalisation. Data analysis was performed using chemometric analysis employed by MetaboAnalyst 4.0 collection of statistical and machine learning algorithms that are highly robust for multidimensional data analysis. Initially, unsupervised multivariate statistical methods such as principal component analysis (PCA) was performed to explore the structure of the data (trends, groupings), allowing the identification of any similarities or differences between and within the samples. For quantitative analysis and biological interpretation, hierarchical cluster analysis (HCA) was performed and (Pearson's correlation distance measure) visualised using heatmaps, boxplots and pathway analysis using MetaboAnalyst 4.0. To globally visualise the metabolite changes in the targeted metabolites, MetaMapp (http://metamapp.fiehnlab.ucdavis.edu/)^96^ was used to compute correlation networks, which were visualized using Cytoscape v3.8.1^96^.

### DNA extraction and quantification of global DNA methylation

Genomic DNA (gDNA) was extracted from leaf samples (control and treatment groups) that were stored at − 80 °C using a modified Cetyltrimethylammonium bromide (CTAB) method. gDNA extraction was performed using 500 mg of leaf tissue, which was ground into a fine powder using liquid nitrogen. This plant material was added to 500 μL of extraction buffer (2% w/v CTAB, 2% w/v polyvinylpyrrolidone (PVP), 0.5 M ethylenediaminetetraacetic acid (EDTA), 5 M sodium chloride (NaCl), 100 mM tris(hydroxymethyl)aminomethane-hydrochloride (Tris–HCl) pH 8.0 and 0.2% v/v β-mercaptoethanol) and incubated at 65 °C for 60 min. Following incubation, 500 μL of chloroform : isoamyl alcohol (24:1) was added to each sample and the mixture then centrifuged at 13,000×*g* for 10 min at 4 °C. The aqueous phase was aspirated into a new microcentrifuge tube, to which an equal volume (500 μL) of isopropanol was added to induce DNA precipitation. The mixture was then centrifuged 13,000 × *g* for 10 min at 4 °C. The supernatant was discarded, and the precipitated pellet was washed in 1 mL ice cold 70% ethanol (v/v) and centrifuged at 12,000×*g* for 5 min. DNA pellets were dried by heating at 55 °C for 5 min on a heating block and resuspended in TE buffer containing 20 µg/mL of RNase A. The extracted DNA quality and quantity was estimated using the NanoDrop 2000 (NanoDrop Technologies Inc., Rockland, DE, USA), followed by ethidium bromide staining on 1% agarose electrophoresis gels in 1X Tris–acetate-EDTA (TAE) buffer.

Relative quantification of global DNA methylation levels was acquired with an ELISA-based colourimetric assay using the 5-mC DNA ELISA Kit (Zymo Research, Irvine, CA) according to manufacturer's instructions. All samples were assayed in duplicate according to the manufacturer's recommendation and to ensure accurate global DNA methylation detection and quantitation.

### Statistical analysis of global DNA methylation

Statistical analysis was performed using IBM's Statistical Product and Services Solutions software version 26 (SPSS 26, IBM, NY, https://www.ibm.com/analytics/spss-statistics-software)*,* following Pallant^105^ guidelines. The overall significant differences between the groups reported as *p* ≤ 0.05*, *p* ≤ 0.01** and *p* ≤ 0.001*** were analysed using the Kruskal–Wallis test.

### RNA extraction and gene expression study by real-time quantitative PCR (qPCR)

RNA was extracted from 200 mg of *Zea mays* leaves (done for all the treatments**—**Supplementary Table 4) using Direct-zol RNA miniprep plus (Zymo Research, Irvine, CA) according to the manufacturer's recommendation. Concentrations (using A260 = 1 = 40 µg/mL) and purity (using A260/A280 ratio) of extracted RNA samples were determined using the NanoDrop 1000 spectrophotometer (Thermo Fisher Scientific; Waltham, USA) and RNA integrity was assessed by electrophoresis on 1% agarose gel. Five hundred nanograms of the total RNA extracted from each biological replicate per treatment was used for first strand cDNA synthesis (Supplementary Table 7), which was performed using random hexamers and LunaScript RT SuperMix Kit (E3010, New England Biolabs, Massachusetts, USA) in 20 µL reactions, per the manufacturer's recommendation. The synthesised cDNA (1 µL) was used in the second step PCR using LunaScript Universal qPCR Master Mix (M3003, New England Biolabs, Massachusetts, USA), per the manufacturer's recommendations. Reactions (Supplementary Table 8) were performed on a CFX-96 (BioRad, Johannesburg, SA) system, with the thermal cycling conditions as follows: initial denaturation 95 °C for 1 min followed by 40 cycles of 95 °C for 15 s and 61.2 °C for 30 s. The lyophilised primer sets (Integrated DNA Technologies, Coralville, IA) used in this study (Supplementary Table 9), were dissolved in TE buffer (Integrated DNA Technologies, Coralville, IA) to a stock solution of 100 µM and aliquots of 10 µM were prepared in nuclease-free water. Elongation factor 1 alpha (EF1α), and β-tubulin (β-TUB) primer sets were used for normalisation of gene expression which have been reported to be the most stability expressed reference genes under abiotic stress^106^ and 'no template' and ‘no RT’ (Supplementary Table 10) controls were included in each run. Relative quantity (∆Cq) (1) for each sample per gene of interest against control samples was calculated according to the CFX Maestro Software (BioRad, Johannesburg, SA) equations and guidelines (Supplementary—relative quantity).

### H_2_O_2_, ascorbate and malonaldehyde content

The H_2_O_2_ content was assayed according to Brennan and Frenkel^107^. One hundred mg of chilled leaf tissue was macerated in 4 mL cold acetone and the homogenate was filtered through a Whatman No. 1 filter paper. Two mL of this filtrate were treated with 1 mL of titanium reagent (20% titanium tetrachloride in concentrated HCl, 32% v/v) and 1 mL of concentrated ammonia solution to precipitate the titanium-hydroperoxide complex. After centrifugation (at 5000×*g* for 30 min) the precipitate was dissolved in 2 N H_2_SO_4_ and the absorbance was obtained at 415 nm. The H_2_O_2_ content was calculated from a standard curve prepared in a similar way and expressed as μmol.g^−1^ fresh mass (fm). The ascorbate (AsA) content was assayed according to the method described by Hodges et al.^108^. To determine the total ascorbate content, 200 µL of the supernatant (from homogenisation of 5 g of fresh weight leaf tissue and centrifuged) was added to 500 µL of a 150 mM K_2_PO_4_ buffer solution (pH 7.0) containing 5 mM EDTA and 100 µL of 10 mM dithiothreitol (DTT) to reduce DHA to AsA. The reaction was allowed to continue for 15 min, after which 100 µL of a 0.5% N-ethylmaleimide solution was added to the reaction mixture at 25 °C to quench the excess DTT. The solution was coloured by adding 400 µL of a 44% *o*-phosphoric acid solution, 400 µL of a 10% trichloroacetic acid (TCA) solution, 200 µL of 30 g.L^−1^ FeCl_3_ solution and 400 µL of α-dipyridyl in 70% (v/v) ethanol solution. The solution was kept at 40 °C for 60 min after which the absorbance at 525 nm was measured spectrophotometrically. The concentration was estimated by using a standard curve. Malonaldehyde was measured spectrophotometrically using the thiobarbituric (TBA) method according to Dhindsa et al.^109^. A volume of 2 mL of the extract was added to a solution containing 1 mL of a 20% trichloroacetic acid (TCA) and 0.5% thiobarbituric acid (TBA). The mixture was heated in a water bath at 95 °C for 30 min. The solution was allowed to cool to room temperature and centrifuged at 14,000 rpm for 10 min. The absorbance was read at 532 nm and the non-specific absorbance at 600 nm and 440 nm was subtracted from the measured absorbance value. The MDA content was calculated by using an extinction coefficient of 155 mM^−1^ cm^−1^.

### Extraction of antioxidant enzymes and enzyme activity analysis

Frozen (− 80 °C) leaf tissue (0.5 g) was homogenised in 1.5 mL of a 50 mM potassium phosphate buffer (pH 7.8) containing 1 mM EDTA, 1 mM dithiotreitol (DTT) and 2% (w/v) polyvinylpyrrolidone (PVP) using a chilled mortar and pestle kept on ice. The homogenate was centrifuged at 15,000×*g* at 4 °C for 30 min. The clear supernatant was used for superoxide dismutase enzyme assays. For measuring ascorbate peroxidase activity, the tissue was separately ground in 50 mM PBS (pH 7.8) supplemented with 2 mM ascorbate, 1 mM EDTA, 1 mM DTT and 2% (w/v) PVP. All assays were done at 25 °C. Ascorbate peroxidase (APX) (EC 1.11.1.11) was assayed according to Nakano and Asada^110^. This was done by taking 3 mL of a reaction mixture (described above) containing 50 mM potassium phosphate buffer (pH 7.0), 0.1 mM EDTA, 0.5 mM ascorbate, 0.1 mM H_2_O_2_ and 0.1 mL enzyme extract and following the hydrogen peroxide-dependent oxidation of ascorbate by measuring the decrease in the absorbance at 290 nm (E = 2.8 mM^−1^ cm^−1^). Ascorbate peroxidase activity was expressed as µmol ascorbate oxidized.min^−1^ mg^−1^ protein. Superoxide dismutase (EC 1.15.1.1) activity was assayed using the kit (A001-1) provided by Elabscience, Total superoxide dismutase (T-SOD) activity assay kit, WST-1 method, which is based on the method described by Beyer and Friedovich^111^. One unit of SOD activity was defined as the amount of enzyme required for 1 mg tissue proteins in 1 mL of a reaction mixture to raise SOD inhibition rates to 50% at 550 nm, expressed as µg.mg^−1^ protein. Catalase (EC 1.11.1.6) activity was assayed using an assay kit provided by Elabscience, CAT-activity kit. Catalase activity was estimated as the amount of enzyme that decomposes 1 µmol H_2_O_2_ at 405 nm sec^−1^ in 1 mg fresh tissue proteins, expressed as µg.mg^−1^ protein.

## Supplementary Information

Supplementary Information.
