# Supplementary material for: Molecular mechanisms associated with microbial biostimulant-mediated growth enhancement, priming and drought stress tolerance in maize plants
Source: Sci Rep. 2022 Jun 21;12:10450. doi: 10.1038/s41598-022-14570-7 (PMC9213556; doi:10.1038/s41598-022-14570-7)
Supplement: Supplementary file 1 — Supplementary Information. [file 41598_2022_14570_MOESM1_ESM.pdf]

Supplementary Materials

The supplementary figures and tables are provided as additional information to support the main results reported in this study. All the experimental raw data, figures and tables that are not included in this manuscript are available from the corresponding author, Dr. Fidele Tugizimana, Department of Biochemistry at the University of Johannesburg.

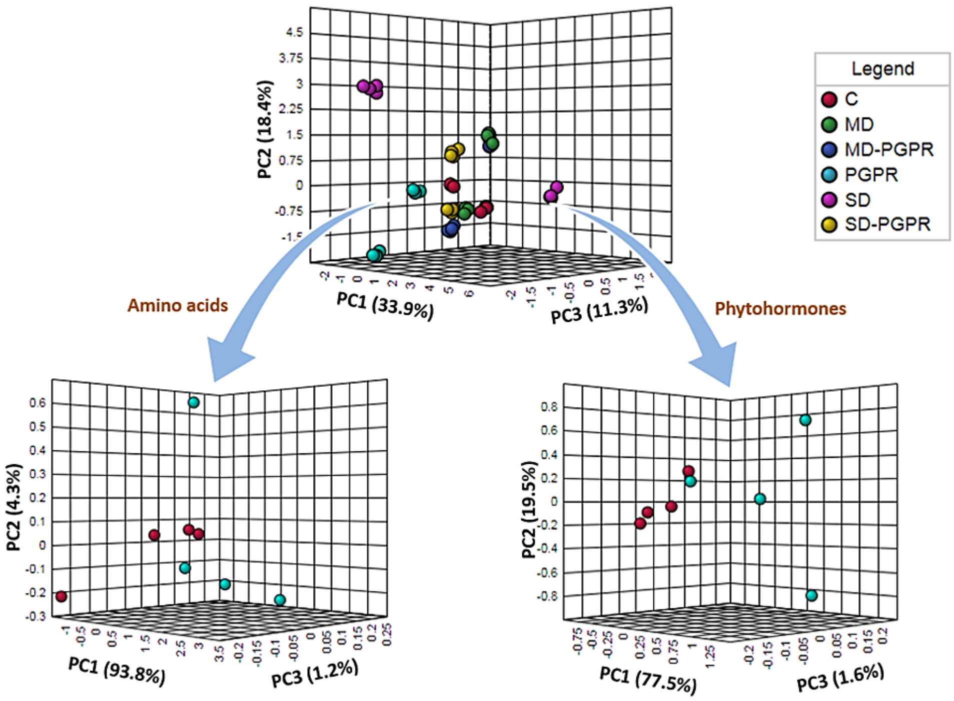

Fig. 1: **PCA modelling showing the overall structure of data**. A 3D scores scatter plot of the PCA model of all the metabolite classes and treatments: model explains 92.4% (5-components) of the total variation in the log-transformed and *Pareto* scaled data at 4 and 6 weeks after emergence (WAE) leading to PCA modelling of well-watered plants (C) and well-watered plants with PGRP (PGPR) at 4 WAE for amino acids and phytohormones. The model provided an overview of the data allowing the identification of sample grouping and natural clustering in multivariate space. The global view shows treatment related groupings, and a further zoom in shows the control samples (red) grouping away from the PGPR treated samples (blue) under well-watered conditions. Abbreviations: C; control, MD; mild drought, MD-PGPR; mild drought and PGPR, SD; severe drought and SD-PGPR; severe drought and PGPR.

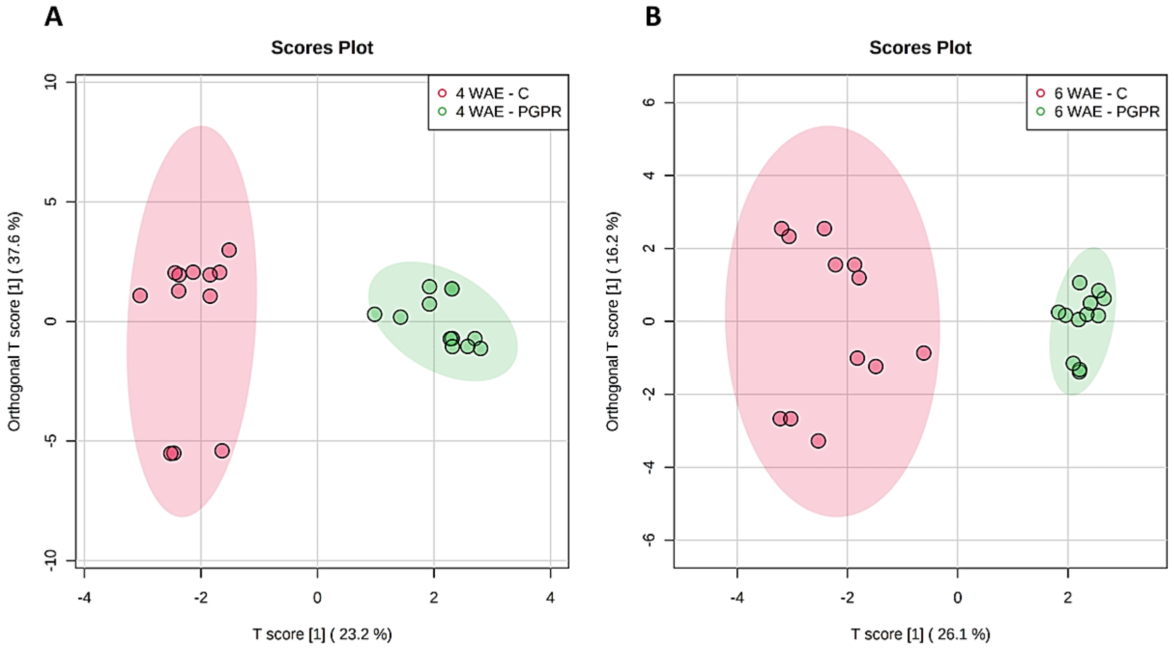

Fig. 2: **OPLS-DA modelling of phytohormones and amino acids under control conditions and PGPR treatment**. (**A**) 4 WAE and (**B**) 6 WAE. The scores plot shows a clear separation of the control (red) vs. PGPR-treated plants (green) under well-watered conditions, with *p*-values < 0.05 following permutation tests. The evaluation of these models further allowed the extraction of statistically significant variables driving the differentiation between naïve plants and PGPR-treated plants.

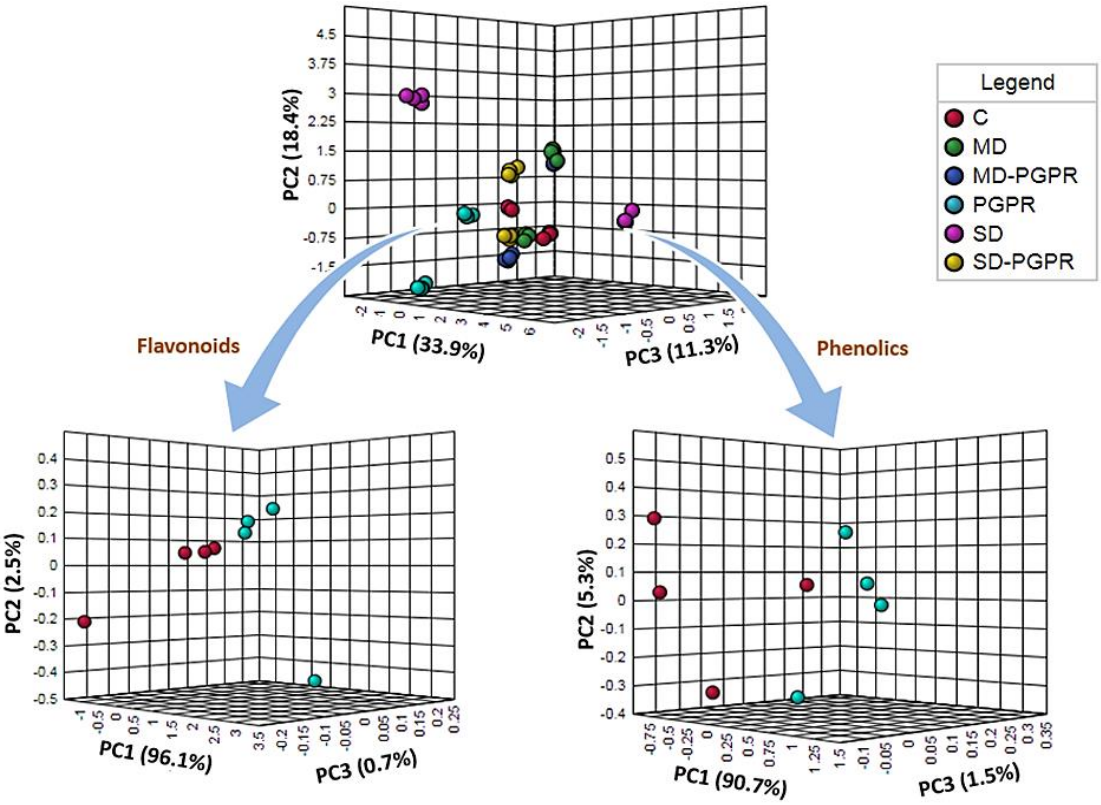

Fig. 3: **PCA modelling showing the overall structure of data**. A 3D scores scatter plot of the PCA model of all the metabolite classes and treatments: model explains 92.4% (5-components) of the total variation in the log-transformed and *Pareto* scaled data at 4 and 6 WAE leading to PCA modelling of well-watered plants (C) and PGRP- treated plants (PGPR) at 4 WAE for flavonoids and phenolics. The model provided an overview of the data allowing the identification of sample grouping and natural clustering in multivariate space. The global view shows treatment related groupings, and a further zoom in shows the control samples (red) grouping away from the PGPR treated samples (blue) under well-watered conditions. Abbreviations: C; control, MD; mild drought, MD-PGPR; mild drought and PGPR, SD; severe drought and SD-PGPR; severe drought and PGPR.

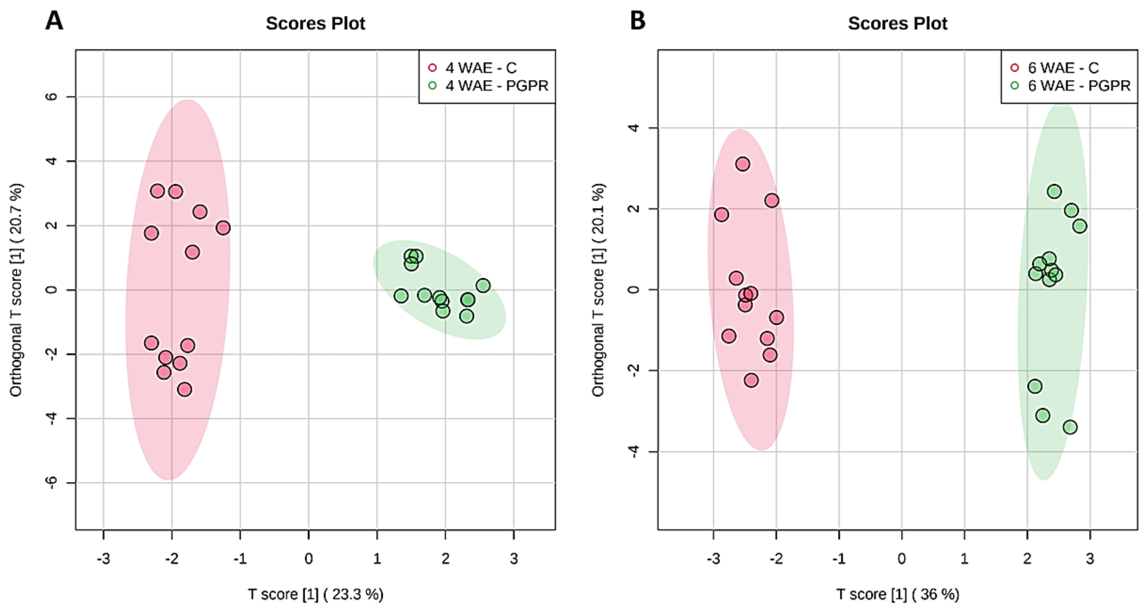

Fig. 4: **OPLS-DA modelling of flavonoids and phenolic acids under control conditions and PGPR treatment**. (**A**) 4 WAE and (**B**) 6 WAE. The scores plot shows a clear separation of the control (red) *vs*. PGPR-treated plants (green) under well-watered conditions, with *p*-values < 0.05 following permutation tests. The evaluation of these models further allowed the extraction of statistically significant variables driving the differentiation between naïve plants and PGPR-treated plants.

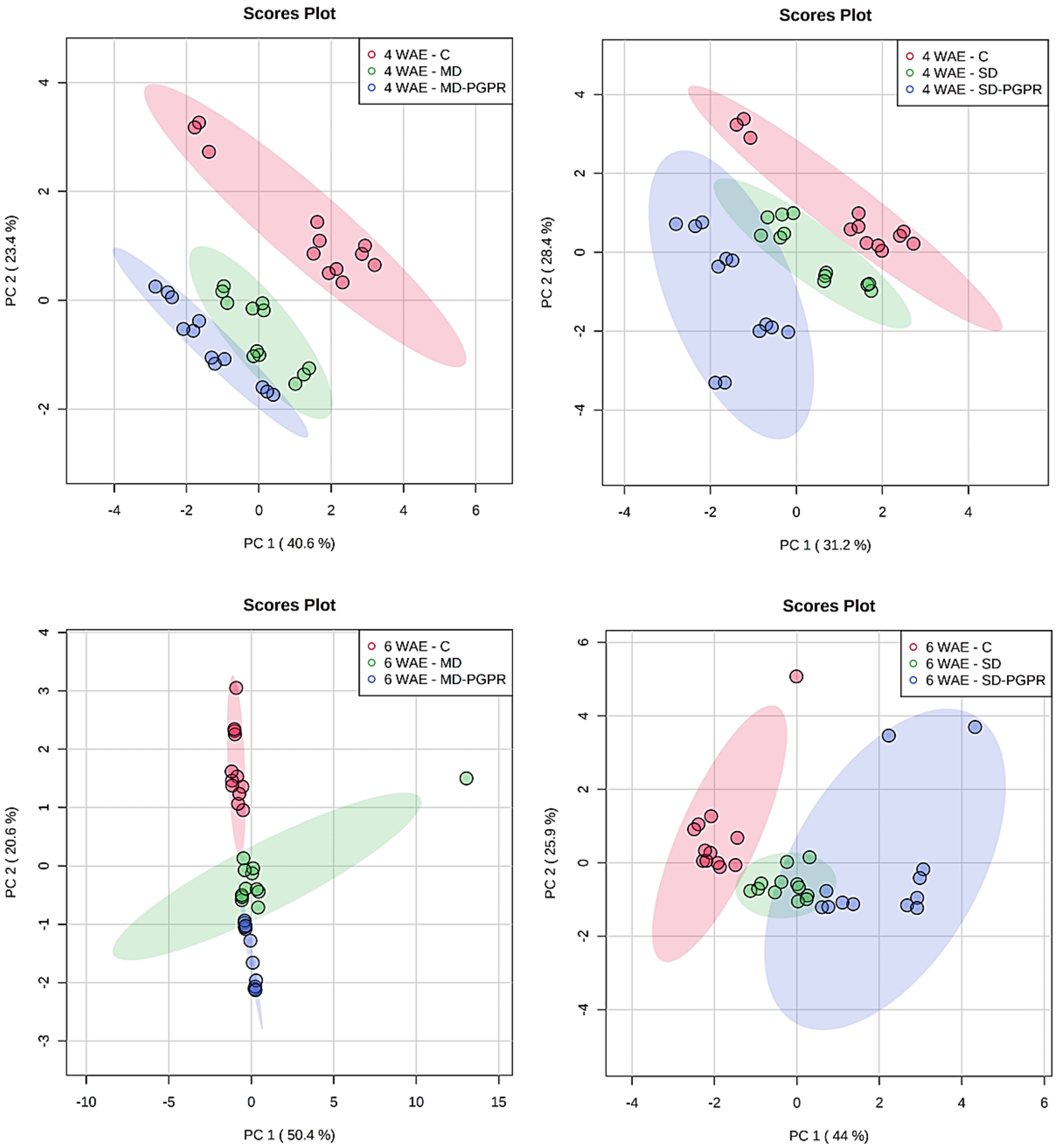

Fig. 5: **PCA scores plot showing treatment related groupings**. PCA analysis of amino acids and phytohormones under control, mild drought, severe drought, mild drought with PGPR and severe drought with PGPR conditions at 4 and 6 WAE. The models provide an overview of the data allowing the identification of sample grouping and natural clustering in multivariate space under drought stress conditions. Distinct treatment related groupings were observed between the control (C), mild drought, mild drought with PGPR (MD-PGPR), severe drought (SD) and severe drought with PGPR (SD-PGPR) treatments.

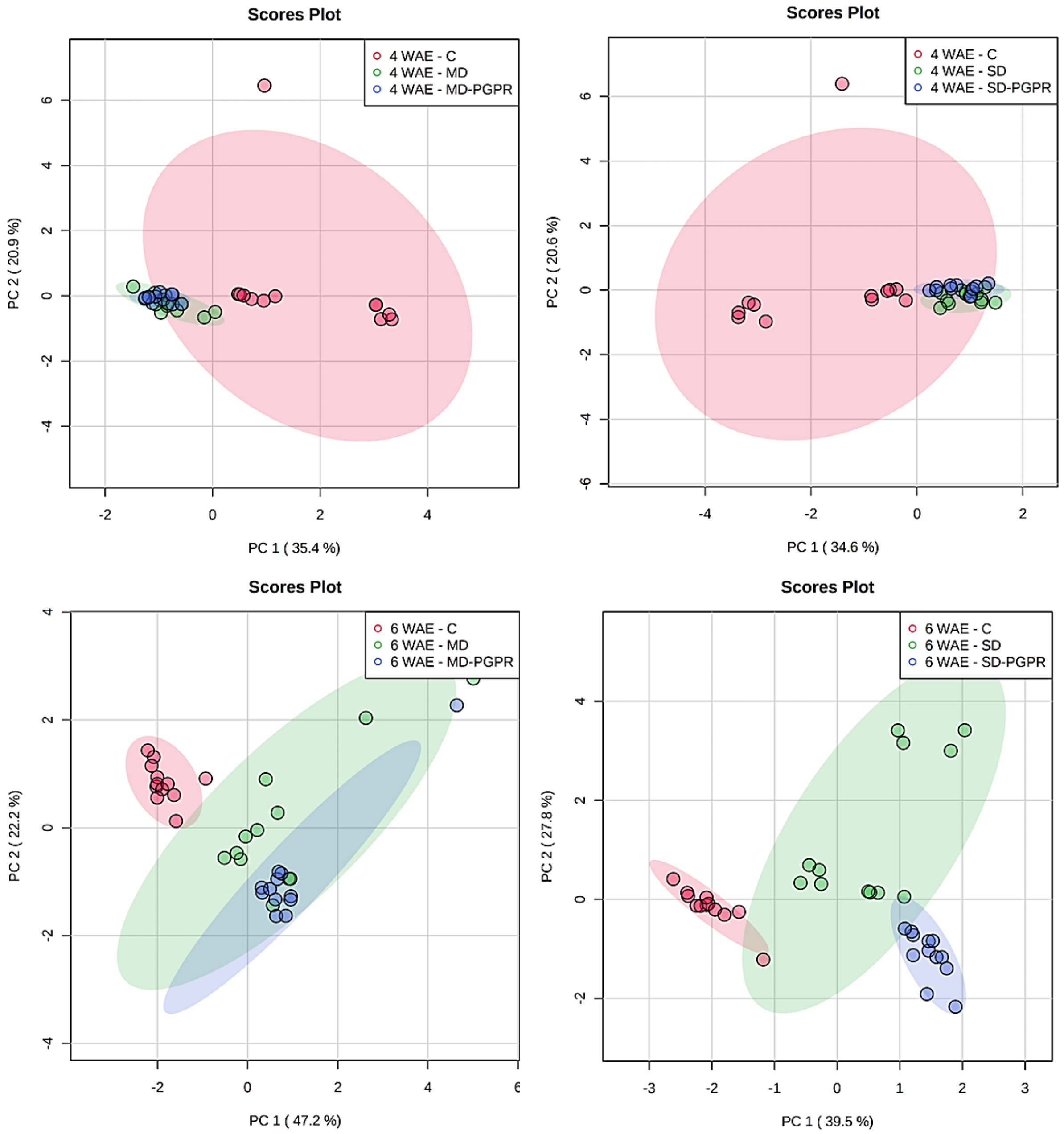

Fig. 6: **PCA scores plot showing treatment related groupings**. PCA analysis of flavonoids and phenolic acids under control, mild drought, severe drought, mild drought with PGPR and severe drought with PGPR conditions at 4 and 6 WAE. The models provide an overview of the data allowing the identification of sample grouping and natural clustering in multivariate space under drought stress conditions. Distinct treatment related groupings were observed between the control (C), mild drought, mild drought with PGPR (MD-PGPR), severe drought (SD) and severe drought with PGPR (SD-PGPR) treatments.

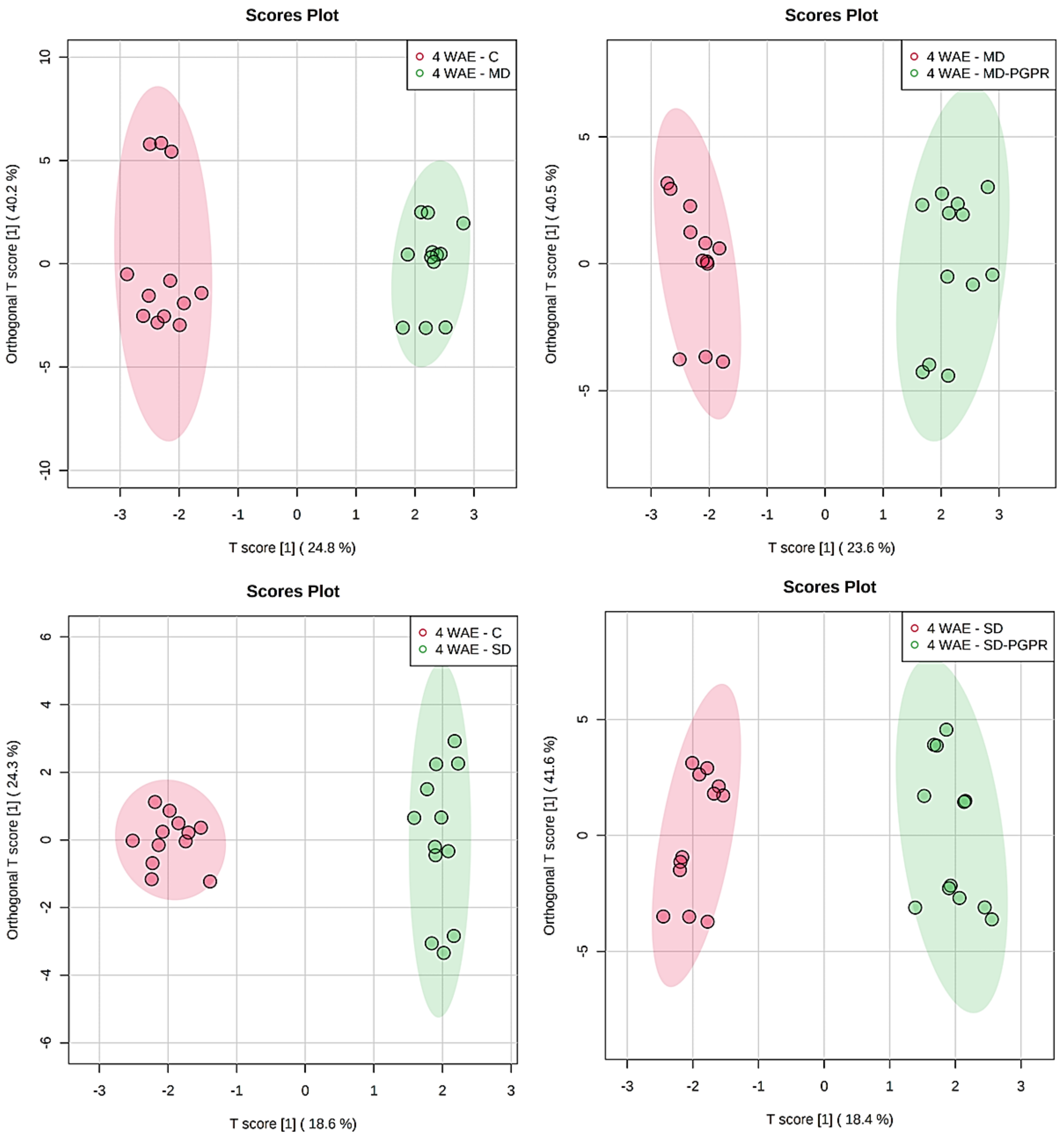

Fig. 7: **OPLS-DA modelling of amino acids and phytohormones under control conditions *versus* mild drought and severe drought stress together with PGPR treatment at 4 WAE**. The scores plot shows a clear separation of the control (red) *vs*. mild drought (MD) and severe drought (SD) (green) conditions and mild drought (MD) and severe drought (SD) (red) *vs*. drought stress conditions with PGPR treatment (green). The evaluation of these models further allowed the extraction of statistically significant variables driving the differentiation between plants under drought stress conditions and PGPR-treated-drought stressed plants.

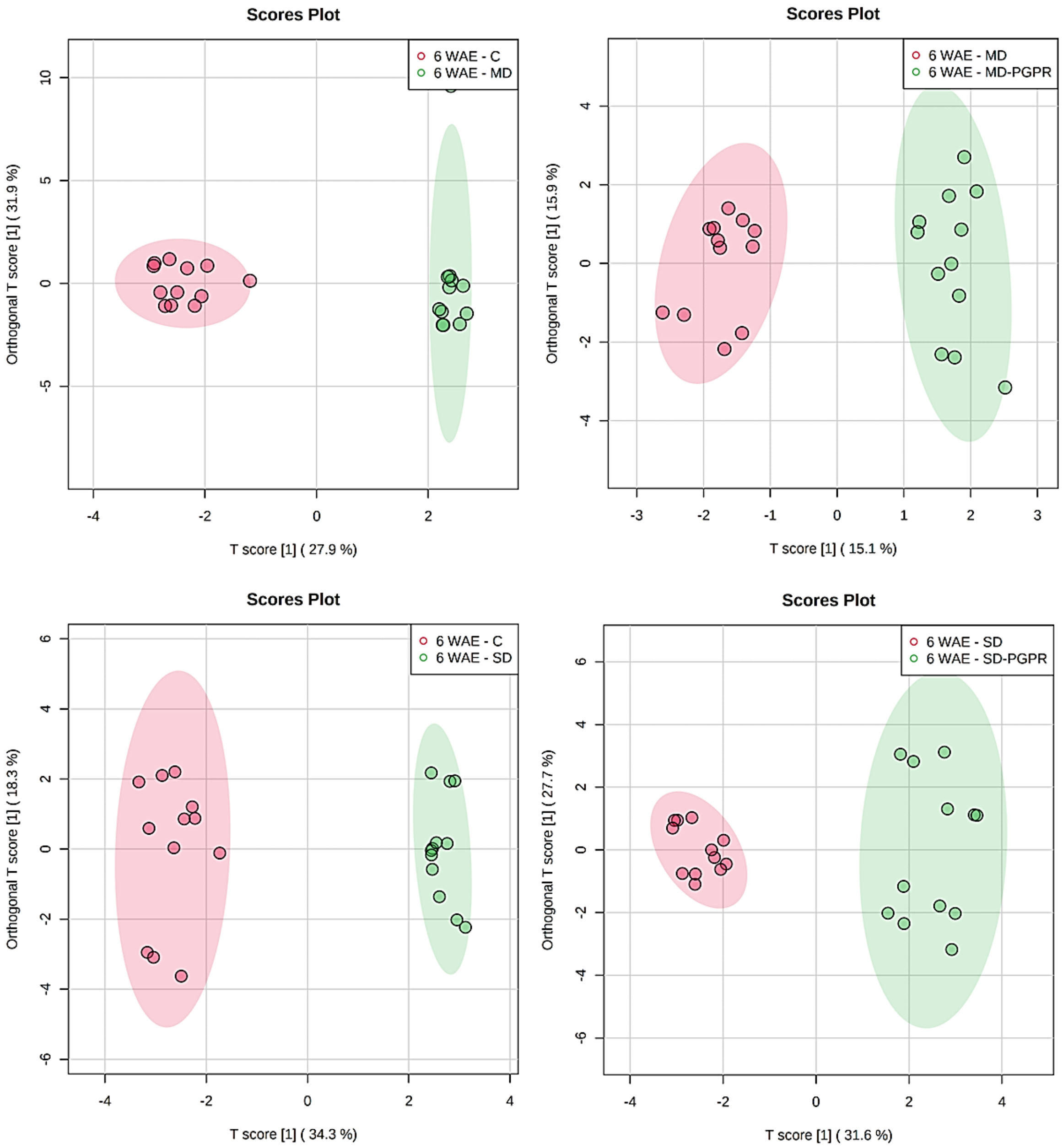

Fig. 8: **OPLS-DA modelling of amino acids and phytohormones under control conditions *versus* mild drought and severe drought stress together with PGPR treatment at 6 WAE**. The scores plot shows a clear separation of the control (red) *vs*. mild drought (MD) and severe drought (SD) (green) conditions and mild drought (MD) and severe drought (SD) (red) *vs*. drought stress conditions with PGPR treatment (green). The evaluation of these models further allowed the extraction of statistically significant variables driving the differentiation between plants under drought stress conditions and PGPR-treated-drought stressed plants.

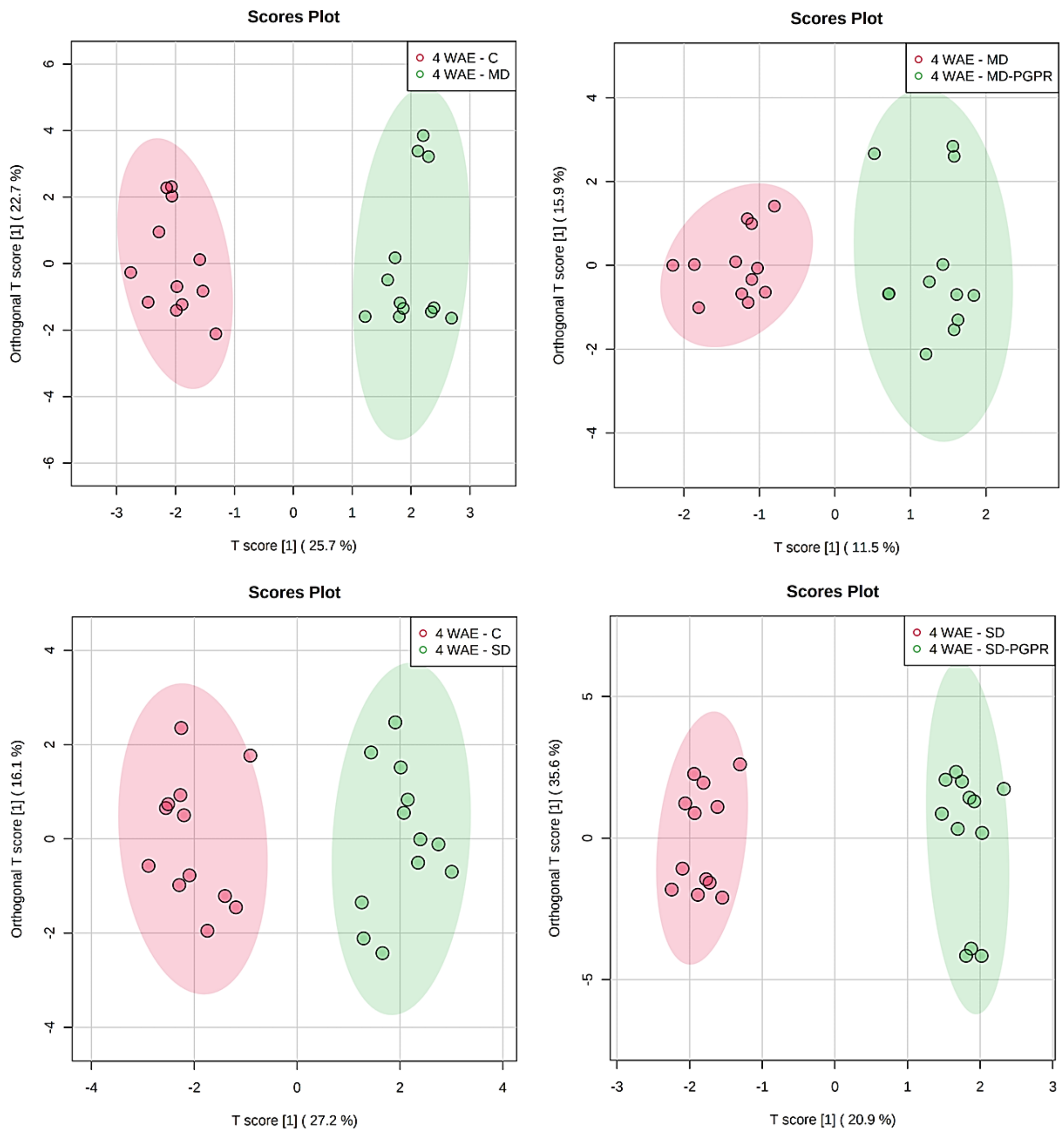

Fig. 9: **OPLS-DA modelling of flavonoids and phenolic acids under control conditions *versus* mild drought and severe drought stress together with PGPR treatment at 4 WAE.** The scores plot shows a clear separation of the control (red) *vs*. mild drought (MD) and severe drought (SD) (green) conditions and mild drought (MD) and severe drought (SD) (red) *vs*. drought stress conditions with PGPR treatment (green). The evaluation of these models further allowed the extraction of statistically significant variables driving the differentiation between plants under drought stress conditions and PGPR-treated-drought stressed plants.

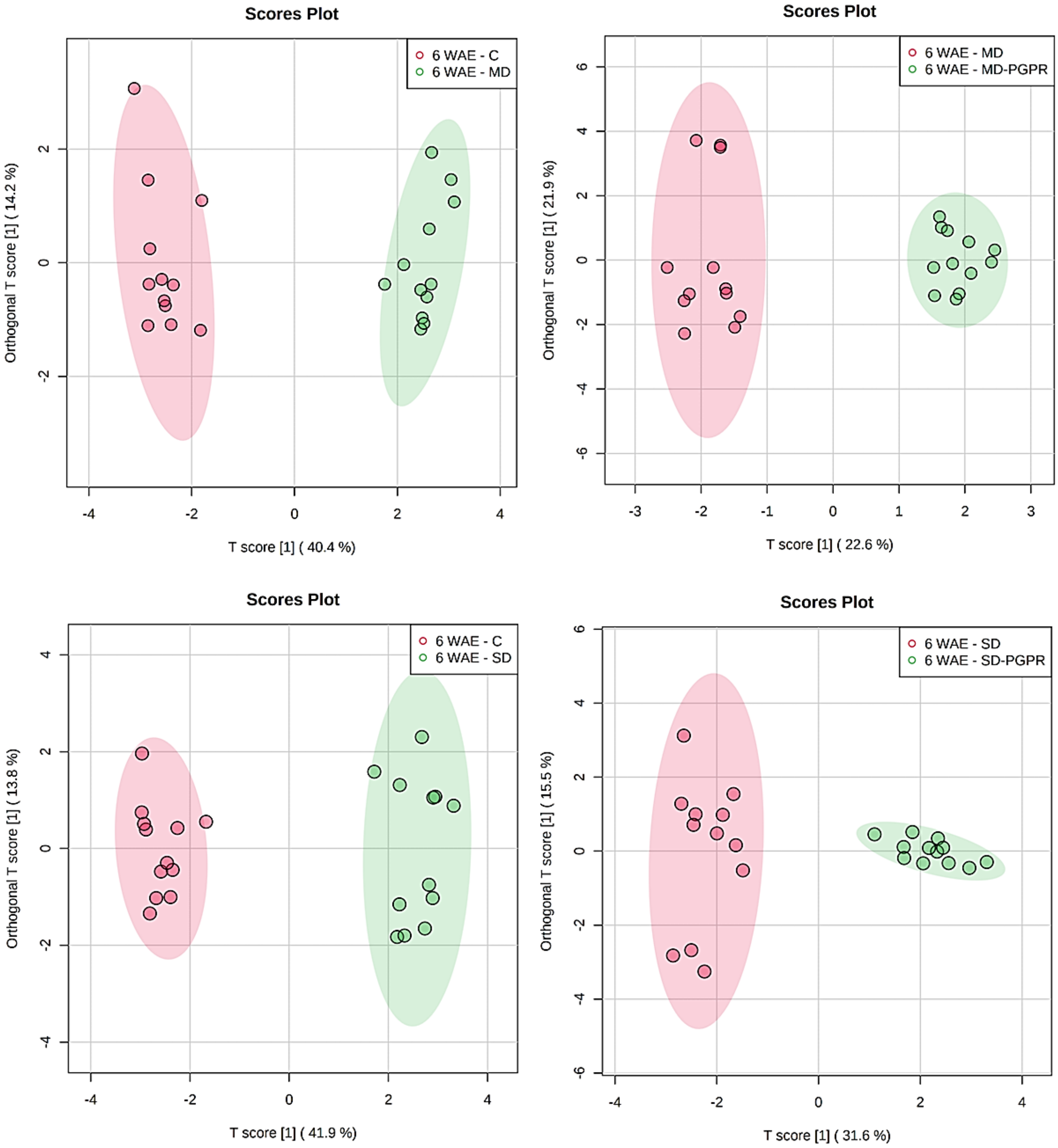

Fig. 10: **OPLS-DA modelling of flavonoids and phenolic acids under control conditions *versus* mild drought and severe drought stress together with PGPR treatment at 6 WAE**. The scores plot shows a clear separation of the control (red) *vs*. mild drought (MD) and severe drought (SD) (green) conditions and mild drought (MD) and severe drought (SD) (red) *vs*. drought stress conditions with PGPR treatment (green). The evaluation of these models further allowed the extraction of statistically significant variables driving the differentiation between plants under drought stress conditions and PGPR-treated-drought stressed plants.

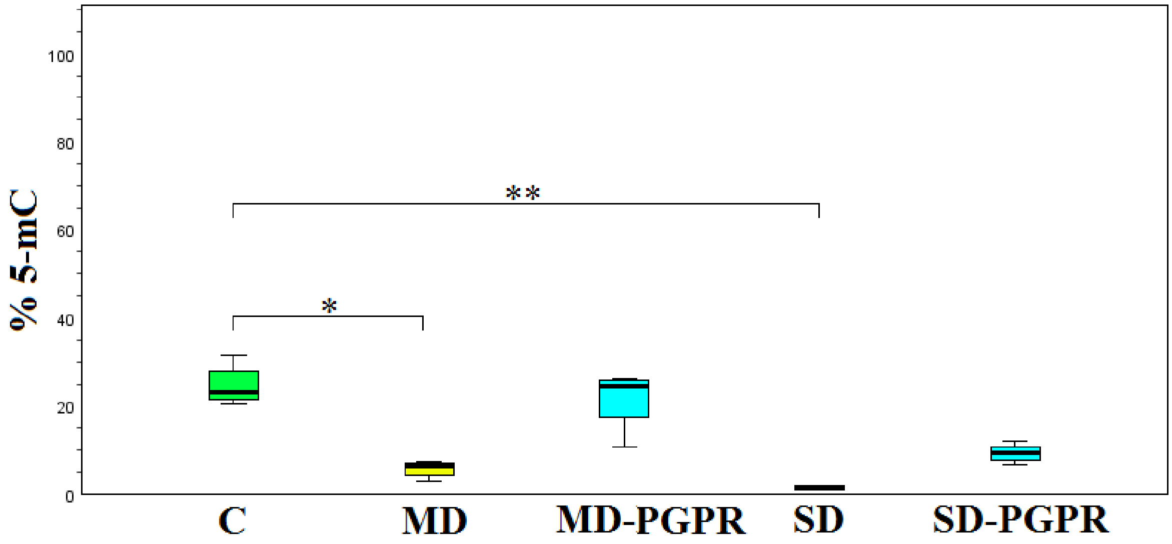

Fig. 11: **Global DNA methylation levels in control, drought stress and drought-stress PGPR treatment conditions in maize leaves**. Genomic DNA from leaves in the different conditions were used to determine the relative global DNA methylation using a commercial ELISA-based kit. The methylation values represented in the plot correspond to the percentage methylation of the samples relative to the methylated control DNA samples supplied with the kit. The horizontal line within a box-plot represents the median. The lower and upper edges of boxes show the 25th and 75th percentiles, respectively. Whiskers represent the maximum and minimum values. **Abbreviations**: C, control; PGPR, plant growth-promoting rhizobacteria; MD, mild drought and SD, severe drought. Statistical significance findings were reported as $p \leq 0.05$* and $p \leq 0.01$**.

Table 1: Significant metabolic pathways involved in response to PGRP treatment in non-stressed *Zea mays*.

No	Pathway	Total	Hits	FDR	Impact
**1**	Glycine, serine and threonine metabolism	33	6	4.13E-07	0.51346
**2**	Phenylalanine metabolism	12	1	7.85E-05	0.42308
**3**	Isoquinoline alkaloid biosynthesis	6	1	6.43E-05	0.41176
**4**	Cysteine and methionine metabolism	46	5	1.15E-08	0.20121
**5**	Tyrosine metabolism	18	1	6.43E-05	0.16757
**6**	Arginine and proline metabolism	28	2	0.000188	0.1601
**7**	Alanine, aspartate and glutamate metabolism	22	2	5.99E-05	0.1259
**8**	Flavonoid biosynthesis	47	3	5.13E-07	0.12294
**9**	Glyoxylate and dicarboxylate metabolism	29	2	4.13E-07	0.11945
**10**	Aminoacyl-tRNA biosynthesis	46	12	1.39E-06	0.11111

Table 2: Selected physiological stress markers of the plant cellular milieu. Different letters indicate statistically significant differences between treatments; $p \leq 0.05$.

**Level / Concentration**	**Without PGPR**			**With PGPR**		
	**Control**	**MD**	**SD**	**Control**	**MD**	**SD**
**Non-enzymatic stress markers (µmol. g FW^{-1})**						
Leaf H_2O_2	61.7^{a}	91.2^{a}	137.9^{b}	56.5^{a}	79.7^{a}	101.3^{ab}
Leaf malondialdehyde (MDA)	25.3^{a}	34.6^{a}	63.9^{b}	23.7^{a}	28.9^{a}	42.1^{c}

Leaf ascorbate (AsA)	227.5^{a}	329.3^{b}	598.1^{c}	241.1^{a}	409.7^{d}	634.8^{c}
Enzyme activities (μmol. $min^{-1}.mg^{-1}$ protein)						
Superoxide dismutase (SOD)	54.7^{a}	67.9^{b}	89.8^{c}	61.2^{ab}	91.9^{c}	118.6^{d}
Ascorbate peroxidase (APX)	40.4^{a}	46.7^{b}	59.8^{c}	43.6^{ab}	49.8^{b}	72.7^{d}
Catalase (CAT)	4.1^{a}	7.6^{b}	11.5^{c}	4.4^{a}	8.2^{b}	15.4^{d}

Table 3: Significant metabolic pathways involved in response to PGRP treatment in drought stressed *Zea mays*.

**Pathway**	**Total**	**Expected**	**Hits**	**FDR**	**Impact**
Phenylalanine metabolism	12	0.11119	1	0.54361	0.42308
Isoquinoline alkaloid biosynthesis	6	0.055595	1	0.36926	0.41176
Glycine, serine and threonine metabolism	33	0.30577	4	0.007438	0.33218
Cysteine and methionine metabolism	46	0.42623	3	0.14242	0.17729
Tryptophan metabolism	23	0.21311	1	0.73763	0.17241
Tyrosine metabolism	18	0.16679	1	0.61412	0.16757
Tryptophan metabolism	23	0.11475	1	1	0.41379
Flavonoid biosynthesis	47	0.134	3	0.013099	0.12294
Flavone and flavonol biosynthesis	12	0.034212	2	0.018944	0
Phenylalanine, tyrosine and tryptophan biosynthesis	22	0.12545	1	1	0.08008
Phenylpropanoid biosynthesis	35	0.19957	2	1	0.03391

Table 4: Description of treatment conditions used to study the effect of PGPR.

**Treatment**	**PGPR rate of 2 billion cfu per ml ($L\ ha^{-1}$)**	**Treatment description**
**T1**	2	Well-watered with biostimulant (PGPR)
**T2**	0	Well-watered without biostimulant (Control; C)
**T3**	2	Mild drought with biostimulant (MD-PGPR)
**T4**	2	Severe drought with biostimulant (SD-PGPR)
**T5**	0	Mild drought without biostimulant (MD)
**T6**	0	Severe drought without biostimulant (SD)

Table 5: Development and optimisation of MRM parameters by direct infusion and optimisation of the collision energy (CE) for each compound transition using the MRM optimisation method tool.

Compound name	Rt (min)	Ion mode	m/z	Transition	CE (eV)	Quadrupole 1 (V)	Quadrupole 3 (V)	Dwell time (ms)
**Amino acids**								
Proline (Pro)	1.50	[M+H]+	116.20	116.20>70.15	-18.0	-14.0	-11.0	17.0
				116.20>43.10	-28.0	-13.0	-15.0	17.0
Cysteine (Cys)	1.31	[M+H]+	241.20	241.20>151.90	-14.0	-12.0	-15.0	37.0
Serine (Ser)	1.34	[M+H]+	106.20	106.20>59.95	-13.0	-12.0	-10.0	17.0
				106.20>88.10	-13.0	-12.0	-18.0	17.0
Alanine (Ala)	1.40	[M+H]+	90.20	90.20>44.05	-13.0	-10.0	-15.0	17.0
				90.20>44.90	-30.0	-18.0	-16.0	17.0
Threonine (Thr)	1.40	[M+H]+	120.20	120.20>56.05	-16.0	-13.0	-20.0	17.0
				120.20>74.10	-12.0	-13.0	-28.0	17.0
Aspartic acid (Asp)	1.41	[M+H]+	134.05	134.05>74.10	-15.0	-10.0	-13.0	37.0
Valine (Val)	1.73	[M+H]+	118.20	118.20>72.10	-12.0	-14.0	-12.0	17.0
				118.20> 55.05	-23.0	-14.0	-23.0	17.0
Methionine (Met)	2.14	[M+H]+	150.20	150.20>60.90	-17.0	-10.0	-20.0	17.0
				150.20>56.10	-24.0	-11.0	-24.0	17.0
Tyrosine (Tyr)	3.29	[M+H]+	182.00	182.0>136.10	-14.0	-13.0	-24.0	37.0
Phenylalanine (Phe)	5.93	[M+H]+	166.00	166.00>120.10	-14.0	-12.0	-21.0	131.0
Tryptophan (Trp)	6.77	[M+H]+	205.20	205.20>188.05	-11.0	-14.0	-19.0	64.0
				205.20>146.10	-17.0	-14.0	-14.0	64.0
Glycine (Gly)	1.40	[M+H]+	76.10	76.10>76.10	-13.0			100
Betaine* (Osmolyte) (Bet)	1.55	[M+H]+	118.05	118.05	-25.0	-	-	100
**Hormones**								

N-hydorxyethylpthalimide* (NHP)	30.08	[M+H]+	192.15	192.15>192.15	-10.0	-	-	100
Indole-3-carboxyaldehyde* (ICAld)	16.60	[M+H]+	146.05	146.05	-25.0	-	-	100
Indole-3-carboxylic acid* (ICA)	22.00	[M+H]+	161.95	161.95>161.95	-15.0	-	-	100
Indole-3-acetic acid (IAA)	26.81	[M+H]+	176.10	176.10>130.10	-15.0	-20.0	-20.0	65.6
				176.10>77.20	-43.0	-12.0	-20.0	65.6
				176.10>103.10	-30.0	-12.0	-22.0	65.6
Zeatin (Zea)	12.98	[M+H]+	220.15	220.15>202.05	-19.0	-10.0	-19.0	100.0
				220.15>136.00	-24.0	-11.0	-24.0	100.0
				220.15>119.00	-34.0	-10.0	-11.0	100.0
Salicylic acid (SA)	22.60	[M-H]-	137.00	137.00>92.95	15.0	20.0	20.0	65.6
				137.00>65.00	28.0	14.0	10.0	65.6
				137.00>75.05	32.0	14.0	27.0	65.6
1-Amino-cyclopropane carboxylic acid (ACC)	1.58	[M+H]+	101.60	101.60>56.20	-14.0	-18.0	-21.0	65.6
				101.60>28.15	-23.0	-18.0	-10.0	65.6
				101.60>30.20	-37.0	-18.0	-30.0	65.6
**Flavonoids**								
Luteoside (Lut)	9.98	[M+H]+	449.0	449.0>287.15	-21.0	-11.0	-19.0	100.0
				449.0>417.15	-9.0	-13.0	-21.0	100.0
				449.0>153.10	-54.0	-11.0	-29.0	100.0
Vicenin 2 (Vic 2)	4.36	[M+H]+	595.0	595.0>324.90	-35.0	-22.0	-22.0	100.0
				595.0>475.25	-17.0	-20.0	-16.0	100.0
				595.0>379.20	-30.0	-22.0	-27.0	100.0
Vicenin 3 (Vic 3)	5.99	[M-H]-	563.0	563.0>353.0	30.0	28.0.	17.0	100.0
				563.0>383.05	34.0	28.0	26.0	100.0
				563.0>473.20	29.0	20.0	14.0	100.0
D-fluorophenylalanine (internal standard)	1.50	[M+H]+	184.0	184.0>138.15	-14.0	-12.0	-26.0	100.0
				184.0>91.15	-30.0	-10.0	-17.0	100.0

				184.0>118.15	-22.0	-11.0	-11.0	100.0
Apigetrin (Apig)	15.04	[M-H]-	431.0	431.0>268.10	35.0	20.0	29.0	100.0
				431.0>269.15	25.0	11.0	29.0	100.0
				431.0>210.90	51.0	15.0	20.0	100.0
Isovitexin (Iso)	6.21	[M-H]-	431.0	431.0>311.15	22.0	20.0	21.0	100.0
				431.0>341.00	22.0	20.0	16.0	100.0
				431.0>283.10	36.0	15.0	29.0	100.0
Vitexin (Vit)	6.22	[M-H]-	431.0	431.0>311.15	21.0	20.0	21.0	100.0
				431.0>283.05	33.0	15.0	19.0	100.0
				431.0>341.20	20.0	20.0	24.0	100.0
Naringenin (Nar)	7.19	[M-H]-	271.0	271.0>151.05	16.0	13.0	26.0	100.0
				271.0>119.10	26.0	13.0	21.0	100.0
				271.0>106.95	24.0	13.0	19.0	100.0
Luteolin (Lutn)	16.80	[M-H]-	285.0	285.0>132.95	34.0	13.0	23.0	100.0
				285.0>151.15	26.0	10.0	29.0	100.0
				285.0>175.15	24.0	10.0	19.0	100.0
Apigenin (Apn)	18.37	[M-H]-	269.0	269.0>117.05	34.0	18.0	19.0	100.0
				269.0>88.95	20.0	28.0	15.0	100.0
				269.0>151.10	23.0	13.0	26.0	100.0
**Phenolics**								
Coumaric acid (Cou)	10.24	[M-H]-	163.0	163.0>119.10	17.0	16.0	22.0	100.0
				163.0>93.05	17.0	30.0	16.0	100.0
				163.0>117.15	18.0	29.0	20.0	100.0
Gallic acid (Gal)	1.92	[M-H]-	169.20	169.20>125.15	20.0	11.0	12.0	100.0
				169.20>79.15	18.0	20.0	15.0	100.0
				169.20>80.95	15.0	12.0	22.0	100.0
Caffeic acid (Caf)	6.75	[M-H]-	179.05	179.05>135.10	18.0	11.0	24.0	100.0
				179.05>134.15	18.0	23.0	23.0	100.0
				179.05>79.0	20.0	19.0	12.0	100.0

Cinnamic acid (Cin)	19.33	[M+H]+	149.10	149.10>77.05	-12.0	-27.0	-28.0	100.0
				149.10>42.90	-17.0	-13.0	-15.0	100.0
				149.10>92.85	-11.0	-16.0	-17.0	100.0
Ferulic acid (Fer)	15.69	[M-H]-	193.20	193.20>134.10	13.0	15.0	29.0	100.0
				193.20>178.30	17.0	17.0	21.0	100.0
				193.20>149.10	13.0	10.0	15.0	100.0
Protocatechuic acid (Pro)	1.95	[M-H]-	153.20	153.20>109.15	16.0	14.0	22.0	100.0
				153.20>108.15	17.0	23.0	23.0	100.0
Shikimic acid (Shi)	0.72	[M-H]-	173.05	173.05>111.20	18.0	12.0	17.0	100.0
				173.05>93.05	15.0	15.0	29.0	100.0
				173.05>105.05	12.0	9.0	19.0	100.0
Syringic acid (Syr)	9.39	[M-H]-	197.0	197.0>182.10	27.0	6.0	20.0	100.0
				197.0>123.10	16.0	25.0	26.0	100.0
				197.0>167.20	15.0	180	18.0	100.0
Coniferyl alcohol (Con)	6.66	[M-H]-	179.20	179.20>134.95	18.0	9.0	29.0	100.0
				179.20>110.90	12.0	10.0	21.0	100.0
				179.20>78.80	13.0	26.0	27.0	100.0

* Single ion monitoring (SIM) was used for quantification of these compounds because they did not fragment.

Table 6: Stepwise gradient elution profile for amino acids, phytohormones, flavonoids and phenolic acids with MilliQ water and 0.1% formic acid (eluent A), and methanol with 0.1% formic acid (eluent B).

**Amino acids**		
**Time (min)**	**Mobile phase A (%)**	**Mobile phase B (%)**
1	98	2
2	95	5
2	90	10
2	50	50
1	98	2
**Phytohormones**		
3	98	2
3	90	10
24	80	20
8	95	5
2	98	2
**Flavonoids**		
2	70	30
10	70	30
18	95	5
1	98	2
**Phenolic acids**		
3	95	5
3	80	20
8	70.2	20.8
3	50	50
7	70	30
5	95	5

Table 7: Reaction components for cDNA synthesis.

**Component**	**20 µL Reaction**	**Final concentration**
LunaScript RT SuperMix (5X)	4 µL	1X
RNA sample	Variable[a]	(up to 1 µg)
Nuclease-free Water	to 20 µL[b]	

[a]The concentration of each RNA sample determined the volume to be added to the reaction, ensuring that the final concentration of the cDNA consisted of 500ng RNA.
[b]The volume of nuclease-free water added was dependent on the volume of the RNA added to the mixture.

Table 8: Reaction components for qPCR analysis.

**Component**	**20 µl Reaction**	**Final Concentration**
Luna Universal qPCR Master Mix	5 µL	1X
10 µM forward primer	0.4 µL	0.25 µM
10 µM reverse primer	0.4 µL	0.25 µM
cDNA products	1 µL	< 4 µL
Nuclease-free water	3.2 µL	

Table 9: Targeted key genes and primer sequences used in this study.

**Gene**	**Primer sequences (5'-3')**	**Amplicon length (bp)**	**Reference**
Δ1-Pyrroline-5-carboxylate synthetase (*P5CS*)	F: GCGAGGAAGTGGGCAAGTGGT<br>R: TTGGGGAGGTGGGGTGGC	250	(Sun *et al.*, 2018)
Flavone synthase type 2 (*FSNII*)	F: CAAGATCGACATGTCGGAGTC<br>R: GCATGGTATCCACATTCTTCG	115	(Righini *et al.*, 2019)
Phenylalanine ammonia-lyase (*PAL*)	F: CGAGGTCAACTCCGTGAACG<br>R: GCTCTGCACGTGGTTGGTGA	318	(Farag *et al.*, 2005)
Dehydration-responsive element binding protein 2 (*DREB2A*)	F: GCAGCCCGGAAGGAAGAA<br>R: GATGACAGCTGCCACTGACGTA	70	(Qin *et al.*, 2007)
Elongation factor 1-alpha (*EF1α* – Ref)	F: TGGGCCTACTGGTCTTACTACTGA<br>R: ACATACCCACGCTTCAGATCCT	135	(Phillips *et al.*, 2018)
Beta-tubulin (*β-TUB* – Ref)	F: CTACCTCACGGCATCTGCTATGT<br>R: GTCACACACACTCGACTTCACG	139	(Phillips *et al.*, 2018)

Table 10: Reaction components for no -RT control reactions.

**Component**	**20 µL Reaction**	**Final Concentration**
No-RT Control Mix (5X)	4 µL	1X
RNA (up to 1 µg)	variable[a]	(up to 1 µg)
Nuclease-free water	to 20 µL[b]	

[a]The concentration of each RNA sample determined the volume to be added to the reaction, ensuring that the final concentration of the cDNA consisted of 500ng RNA.

[b]The volume of nuclease-free water added was dependent on the volume of the RNA added to the mixture.

Relative quantity (ΔCq) (1) for each sample per gene of interest against control samples was calculated according to the CFX Maestro Software (BioRad, Johannesburg, SA) equations and guidelines. Following this, normalized expression (ΔΔCq) (2) of each target gene was calculated using the same software against the two reference genes. Normalised gene expression was expressed as logarithmic fold change and fold change.

$$(1)\ \text{Relative Quantity}_{\text{Sample (GOI)}} = \text{E}_{\text{GOI}}^{(\text{C}_{\text{q(control)}} - \text{C}_{\text{q(sample)}}}$$

$$(2)\ \text{Normalised Expression}_{\text{sample (GOI)}} = \frac{\text{RQ}_{\text{sample (GOI)}}}{(\text{RQ}_{\text{sample (Ref 1)}} \times \text{RQ}_{\text{sample(Ref 2)}})^{\frac{1}{2}}}$$

Where: E = Efficiency; RQ = relative quantity; GOI = gene of interest (one target); Ref = Reference gene.

Statistical analysis and graphical representations were carried out in GraphPad Prism 9.0.0. The Mann-Whitney U test was employed to compare the differences between the different groups (C, PGPR, MD-PGPR and SD-PGPR). Mann-Whitney U test is a non-parametric equivalent of the independent samples t-test and in this study, it was used to complement the non-parametric tests (Kruskal-Wallis tests) employed in the DNA methylation analysis.
